# Circular RNA encoding relaxin-2 as a potential therapy for liver fibrosis

**DOI:** 10.1016/j.omtn.2025.102807

**Published:** 2025-12-18

**Authors:** Jiewen Zhong, Zheyu Zhang, Lixing Xiao, Cheng Wang, Yun Yang, Qinghao Zhang, Zefeng Wang

**Affiliations:** 1 Shanghai Institute of Nutrition and Health, University of Chinese Academy of Sciences, Chinese Academy of Sciences, Shanghai, China; 2 Department of System Biology, School of Life Science, Guangming Advanced Research Institute, Southern University of Science and Technology, Shenzhen, Guangdong, China; 3 CirCode Biomedicine Inc., Shanghai, China

**Keywords:** MT: Clinical Applications, circular RNA, mRNA, relaxin, liver fibrosis, translation

## Abstract

Circular RNAs (circRNAs) have recently emerged as a promising new drug modality with significant therapeutic potential due to their higher stability and lower immunogenicity. Here, we report the development of a circRNA encoding human relaxin-2 (cRLN2), a short peptide hormone with well-established therapeutic potential, to treat liver fibrosis in a mouse model. Compared to the modified linear mRNA, cRLN2 mediated stronger and more prolonged expression of relaxin *in vitro*. In addition, the nanoparticle-mediated delivery of cRLN2 achieved a sustained translation into active relaxin in healthy mice with low immunogenicity. In a mouse model of liver fibrosis, cRLN2 treatment significantly decreased hepatic stellate cell activation and consequently reduced collagen deposition in fibrotic mice, while the treatment by relaxin protein showed limited anti-fibrosis effects. Toxicity evaluation confirmed that cRLN2 exhibits excellent safety and tolerability in mice. Collectively, our findings demonstrate that cRLN2 can efficiently express therapeutic proteins *in vivo* and alleviate liver fibrosis without obvious toxic effects, highlighting the potential of circRNAs as a novel therapeutic platform to treat fibrotic diseases.

## Graphical abstract

**Figure:**
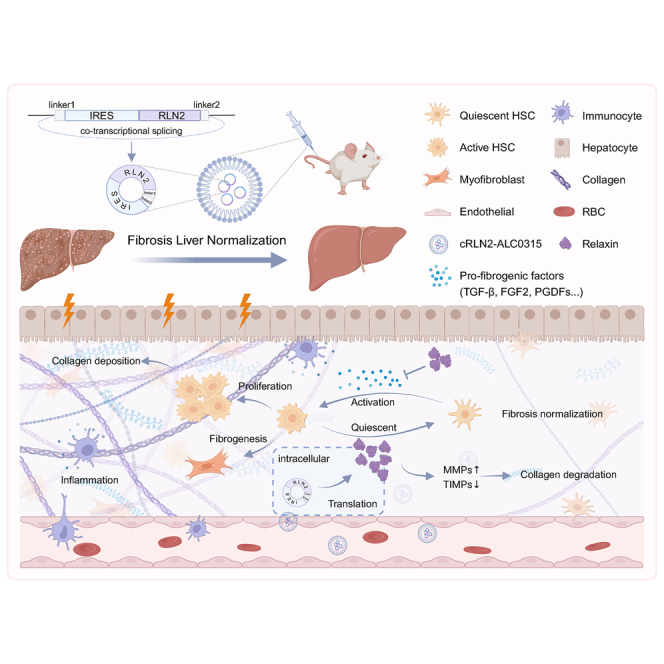

## Teaser

Liver delivery of circRNA-LNP enables sustained and safe expression of human relaxin-2, achieving potent antifibrotic activity under low immunogenicity and excellent tolerability, with efficacy superior to recombinant protein drugs. These findings support circRNAs as a promising platform for protein replacement treatments.

## Introduction

Liver fibrosis is a pathological process characterized by chronic injury or prolonged inflammatory stimulation that activates quiescent hepatic stellate cells (HSCs) through various inflammatory cytokines.^1^ Transforming growth factor (TGF)-β induces fibroblast activation and differentiation into myofibroblasts that secrete extracellular matrix proteins. Canonical TGF-β signaling mobilizes Smad2 and Smad3 transcription factors that control fibrosis by promoting gene expression, leading to excessive deposition of extracellular matrix ([ECM], e.g., collagen), abnormal liver architecture, and functional impairment.^2^ Liver fibrosis represents a common feature of multiple chronic liver diseases and, if left untreated, may progress to cirrhosis or even hepatocellular carcinoma.^3^^,^^4^ It accounts for over 2 million deaths annually (cirrhosis, viral hepatitis, and liver cancer) and accounts for 4% of all deaths worldwide.^5^ The pooled prevalence rates of advanced fibrosis and cirrhosis were 3.3% and 1.3% worldwide, respectively.^6^ The current treatments of liver fibrosis mainly rely on chemical drugs that inhibit essential signaling pathways for fibrosis, such as the activation of TGF-β and the deposition of ECM.^7^^,^^8^ However, targeting these pathways may cause severe side effects in other tissues due to the off-targeting effects. Notably, because liver fibrosis is a chronic and progressive disease, the toxic effect associated with prolonged treatment is an important concern.^9^

The relaxin protein has shown significant therapeutic effects in animal models of various fibrotic diseases across multiple organs including the heart,^10^^,^^11^^,^^12^ liver,^13^^,^^14^ lung,^15^^,^^16^ kidney,^17^^,^^18^^,^^19^ and joint.^20^ The human genome contains three relaxin genes, among which relaxin-2 (RLN2) shares similar antifibrosis effects with mouse relaxin-1 (RLN1) in murine fibrosis models, allowing early-stage development of human relaxin-based drugs to be tested in mouse models.^21^^,^^22^^,^^23^ In liver fibrosis models, relaxin protein interacts with relaxin/insulin-like family peptide receptor 1 (RXFP1), inhibits the release of inflammatory factors and activation of HSCs,^24^^,^^25^^,^^26^ reduces excessive ECM deposition,^27^ upregulates matrix metalloproteinase (MMP) expression,^25^^,^^28^ and promotes the degradation of fibrotic matrices,^26^ thereby achieving therapeutic effects against liver fibrosis. However, as a ∼6-kDa peptide hormone, relaxin has an extremely short half-life, necessitating frequent administrations that severely limit its clinical applications.^29^^,^^30^ Therefore, there is an urgent need to develop a long-acting formulation of the relaxin drug.

The circular RNAs (circRNAs) are a class of covalently closed RNA molecules widely found in eukaryotic cells.^31^ Recent advances in high-throughput technology have demonstrated that circRNAs are highly conserved across species with tissue-specific and developmental stage-specific expression patterns, suggesting their potential as disease biomarkers and therapeutic targets.^32^^,^^33^^,^^34^ Unlike linear mRNAs, circRNAs lack 5′ and 3′ ends, making them resistant to exonuclease degradation and conferring high stability.^35^ While some circRNAs can regulate the expression of other genes by competitively binding to miRNAs or RNA-binding proteins,^36^^,^^37^ many circRNAs have been found to be translated into proteins through cap-independent mechanisms using internal ribosome entry sites (IRES) or IRES-like elements.^38^^,^^39^^,^^40^^,^^41^ This finding suggests that circRNAs may function as a new class of mRNAs capable of expressing therapeutic proteins.^42^ Since RNAs are negatively charged and thus incapable of penetrating cell membranes directly, a carrier system for RNA delivery is required *in vivo*. Lipid nanoparticles (LNPs) are the most widely used carrier system for RNA drugs, which particularly excel in mRNA vaccines.^43^ Currently, four clinically approved LNP formulations have been developed for RNA drugs, including three COVID-19 mRNA vaccines (BNT162b2, mRNA-1273, and ARCT-154)^44^^,^^45^ and a small interfering RNA drug (Onpattro) to treat hereditary transthyretin amyloidosis.^46^ All four LNPs showed preferential delivery of nucleic acid drugs to the liver, likely due to ApoE protein adsorption on the LNP surface, suggesting the therapeutic potential of LNP-based mRNA delivery to liver diseases.^47^^,^^48^

Although circRNAs have emerged as a promising modality for therapeutic protein expression, their current applications remain largely confined to vaccine development,^49^ with protein replacement therapy limited almost exclusively to local administration.^50^ No studies to date have evaluated the use of intravenously (i.v.) delivered therapeutic circRNAs for treating chronic systemic diseases such as liver fibrosis, where local injection is not feasible. Moreover, despite the intrinsic advantages of circRNAs, including markedly enhanced stability and prolonged protein production, there has been no direct comparison between modified mRNA and unmodified circRNA encoding the same therapeutic protein, leaving a critical knowledge gap in understanding their relative pharmacological performance.

In this study, we address these unmet challenges by establishing the first i.v. circRNA-based protein replacement therapy for liver fibrosis. We designed and synthesized circRNAs encoding murine relaxin-1 (cRLN1) and human relaxin-2 (cRLN2) and delivered them to the liver via LNPs to systematically evaluate their expression kinetics, pharmacokinetics (PK), biosafety, and therapeutic efficacy. Studies demonstrated that both cRLN1 and cRLN2 markedly alleviated liver fibrosis in mice, and importantly, unmodified circRNAs exhibited low immunogenicity and excellent tolerability. Compared with modified mRNA, circRNAs achieved higher and more stable expression in cultured cells, and although their *in vivo* expression showed a brief early decline, they maintained prolonged and steady protein production. Moreover, cRLN2 produced antifibrotic effects comparable to those of equimolar doses of modified mRNA. Together, these findings establish circRNA as a powerful platform for *in vivo* therapeutic protein expression and, for the first time, demonstrate the feasibility of i.v. circRNA administration for treating chronic fibrotic diseases, offering new avenues for the development and optimization of circRNA-based therapies.

## Results

### circRNA encoding human relaxin demonstrated anti-fibrotic efficacy in a mouse model

Previously we established an efficient *in vitro* circularization system using an autocatalytic self-splicing group II intron by repositioning intron fragments to both sides of the target sequence and designing 5′/3′ homologous sequences to bring the splice sites into proximity to efficiently produce circRNAs in a co-transcriptional fashion.^51^ The resulting circRNA contains the open reading frame (ORF) of therapeutic protein that undergoes cap-independent translation driven by IRES from coxsackievirus B3 (CVB3), which was found to have strong activity to mediate circRNA translation.^52^ In this study, we engineered circRNA encoding human relaxin-2, which can be translated into cells via the CVB3 IRES to produce active proteins that may potentially exert therapeutic effects for liver fibrosis.

**Figure 1:**
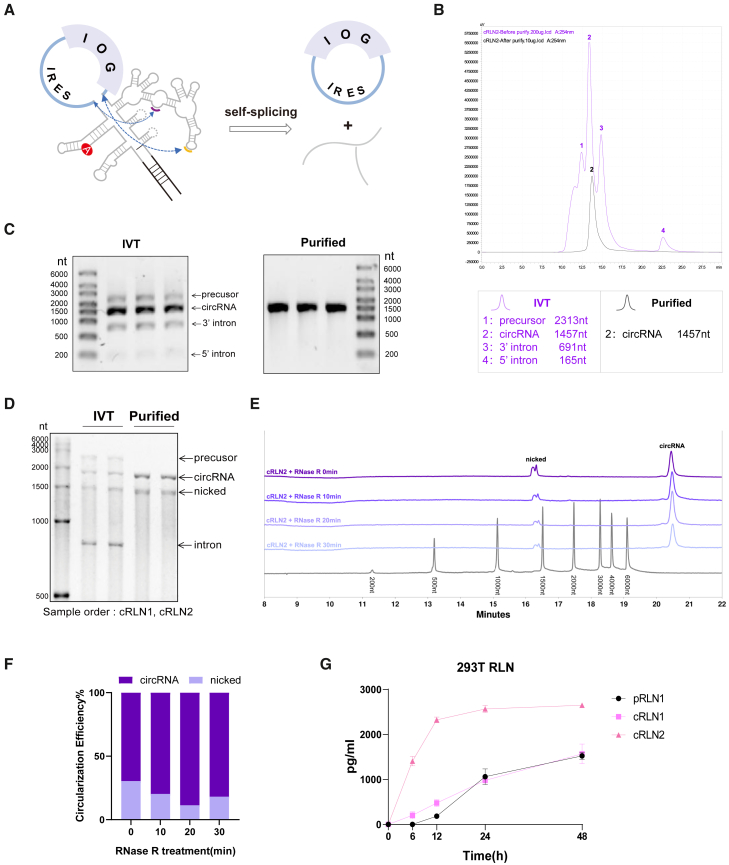
*In vitro* synthesis and purification of circRNAs (A) Schematic diagram of *in vitro* circularization for circRNA production. The autocatalytic self-splicing group II intron was split into two fragments, and customized exons containing IRES and coding region of a gene of interest (GOI) were inserted between the split intron. (B) Representative HPLC chromatograms of circRNAs before and after purification. Pre-purification HPLC shows four major peaks: precursor (2,313 nts), cRLN2 (1,457 nts), 3′ intron (691 nts), and 5′ intron (165 nts). Post-purification HPLC displays a single peak matching the cRLN2 (1,457 nts) stripe. (C) 1% low-melting agarose gel electrophoresis of cRLN before and after purification (*n* = 3 biological replicates). Pre-purification agarose gel shows four major stripes: precursor (2,313 nts), cRLN2 (1,457 nts), 3′ intron (691 nts), and 5′ intron (165 nts). Post-purification agarose gel displays a single circRNAs (1,457 nts) stripe. (D) 4M-1.5% urea-denaturing agarose gel electrophoresis of cRLN1 and cRLN2 before and after purification. The IVT product shows four major stripes: precursor (2,313 nts), circRNAs (>1,457 nts), nicked circRNAs (1,457 nts), and intron (856 nt). Post-purification denaturing agarose gel displays circRNAs and nicked circRNAs stripes. (E) PA800 analysis of cRLN2 circularization efficiency using Thermo RNA Ladder 6000 as reference. Two peaks appear: nicked cRLN2 (1,457 nts) and circular cRLN2 (>6,000 nts). RNase R treatment (0, 10, 20, and 30 min) was applied to remove nicked cRLN2, with circularization rates quantified to determine optimal digestion time. (F) Quantification of peaks of PA800 to reflect circularization efficiency from 1E. (G) RLN2 expression in 293T cells. Equal masses of pRLN1, cRLN1, and cRLN2 were transfected, and relaxin concentration in media was serially measured in 48 h (*n* = 3, biological replicates).

We designed and synthesized the circRNAs by *in vitro* transcription (IVT) (Figure 1A) and further purified them using high-performance liquid chromatography (HPLC) (Figure 1B). Using cRLN2 as an example, pre-purification HPLC shows four major peaks corresponding to four agarose gel stripes (Figure 1C): precursor (2,313 nucleotides [nts]), cRLN2 (1,457 nts), 3′ intron (691 nts), and 5′ intron (165 nts). Post-purification HPLC displays a single peak matching the cRLN2 (1,457 nts) stripe, and closed circular structure was confirmed by Sanger sequencing (Figure S1). Further analysis using urea-denaturing agarose gel electrophoresis revealed the presence of nicked circRNAs before and after purification (Figure 1D). To eliminate nicked circRNAs, RNase R digestion was applied with PA800 analysis, thereby increasing the circularization efficiency to approximately 80% (Figures 1E and 1F). These samples were transfected into 293T cells, with cRLN1 and the plasmid encoding murine relaxin (pRLN1) as controls (Figure 1G). Since relaxin is a secreted protein, we measured its concentration in the cell culture medium at different time points after transfection. The results showed that, within the 48 h after transfection, both cRLN1 and pRLN1 exhibited increased expression of relaxin proteins with no significant differences in expression rates. Additionally, cRLN2 showed higher expression levels than cRLN1 in human-originated 293T cells, confirming that circRNAs can be efficiently translated.

While circRNAs themselves may lack active targeting capabilities, using delivery technologies such as LNPs may enhance drug concentration in specific tissues and minimize side effects on other tissues. Since clinically approved LNPs preferentially deliver nucleic acid drugs to the liver, we tested an LNP formulation containing cationic lipids ALC-0315 and found that it can mediate robust liver expression of circRNAs encoding firefly luciferase (cFluc) after the i.v. injection into healthy mice (Figure S2). Therefore, this LNP formulation was used in the rest of this study to encapsulate different types of mRNA and circRNAs (Table S1). Physicochemical characterization of cRLN1-ALC0315 and cRLN2-ALC0315 using dynamic light scattering revealed that the particles exhibited an encapsulation efficiency exceeding 80%, an average size ranging from 80 to 160 nm, a polydispersity index below 0.24, and a zeta potential of approximately ±15 mV. These results indicate that cRLN1-ALC0315 and cRLN2-ALC0315 had similar physicochemical properties.

**Figure 2:**
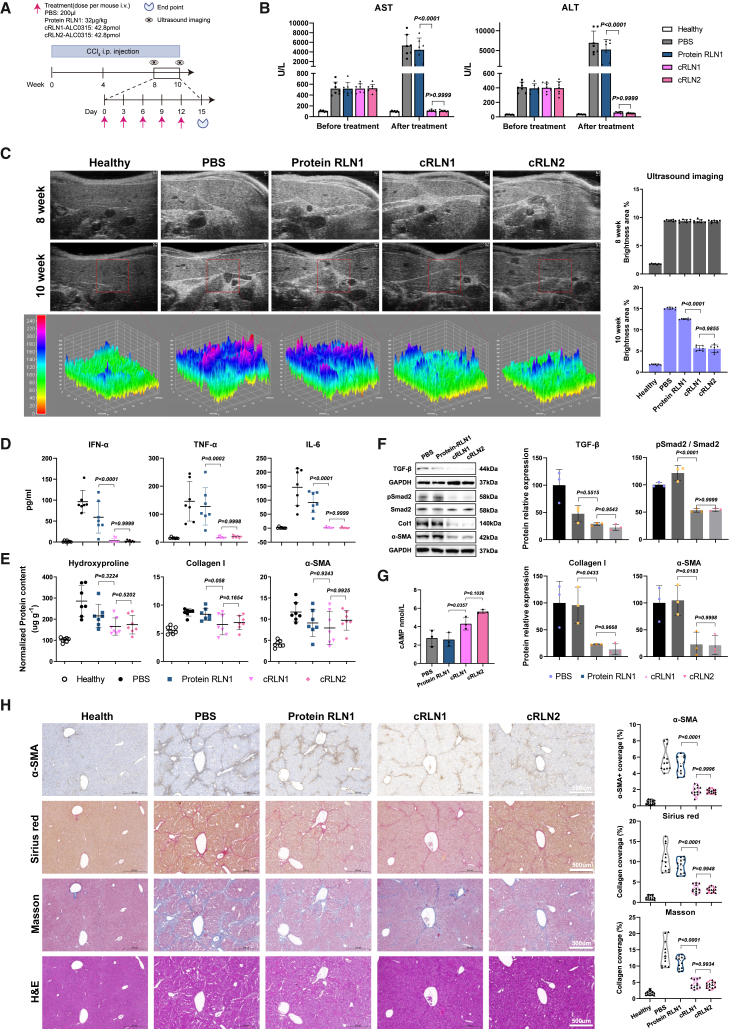
Both human and murine relaxin effectively reversed liver fibrosis in mice (A) Experimental scheme of carbon tetrachloride (CCl_4_)-induced liver fibrosis model with therapeutic interventions: PBS, Protein RLN1 (32 μg/kg), cRLN1, and cRLN2 treatments (42.8 pmol). (B) Serum ALT and AST levels at week 8 (before treatment) and 10 (after treatment) to assess hepatic injury across Healthy, PBS, Protein RLN1, cRLN1, and cRLN2 groups (*n* = 7 mice). (C) Representative *in vivo* ultrasound images of livers at weeks 8 and 10 post-CCl_4_. Fibrosis progression is evidenced by enhanced echogenicity and heterogeneity. 3D surface plots within red dashed boxes (week 10) illustrate echotexture uniformity. Fibrosis severity was quantified by integrated echointensity (bright area) (*n* = 7 mice). (D) Quantification of ELISA measurements for IFN-α, TNF-α, and IL-6 in serum of different treatment groups (*n* = 7 mice). (E) Quantification of ELISA measurements for hydroxyproline, collagen I, and α-SMA in liver tissue (*n* = 7 mice). (F) Western blot analysis of TGF-β (44 kDa), pSmad2 (58 kDa), Smad2 (58 kDa), α-SMA (42 kDa), and collagen I (140 kDa) expression in liver tissues. Data shown are representative of three independent experiments with similar results. Band intensity was quantified using ImageJ (*n* = 3 mice). (G) Quantification of ELISA measurements for cAMP in serum of different treatment groups (*n* = 3 mice). (H) Liver sections showing IHC (α-SMA), sirius red, Masson’s trichrome, and H&E staining. Scale bars, 500 μm. Quantification was performed on four randomly selected fields/mouse (*n* = 3 mice). Both *in vitro* and *in vivo* experiments were repeated twice independently with similar results. Significant differences were assessed using a one-way ANOVA with Tukey test (B–H). Results are presented as mean ± SD from the second repeat. The *p* values for the comparison between each treatment group and the PBS group are shown in Table S5.

We established an experimental liver fibrosis model by intraperitoneally injecting carbon tetrachloride (CCl_4_) into 8-week-old male mice, with liver echogenicity monitored throughout the treatment period (Figure 2A). At week 8 of CCl_4_ administration, the mice showed significantly elevated levels of aspartate aminotransferase (AST) and alanine aminotransferase (ALT) in the bloodstream (Figure 2B), two important enzymes released from the liver during injury. In addition, the liver stiffness was measured by the brightness area of ultrasound images corresponding to acoustic interface reflections. We found that the liver exhibited severe fibrosis as evidenced by a 10-fold increase in the brightness area of ultrasound images compared to Healthy controls (Figure 2C). Two weeks after the treatment, the cRLN1 encoding murine relaxin significantly reduced the liver fibrosis as judged by the brightness area in ultrasound reflections (∼3-fold) and also restored the elevated AST and ALT levels back to normal. Notably, the cRLN2 encoding human relaxin showed comparable therapeutic efficacy to cRLN1 as judged by both ultrasound imaging and ALT and AST levels (Figures 2B and 2C). In comparison, both circRNAs showed superior therapeutic effects than the treatment by the RLN1 protein (*p* value > 0.0001). The *p* values for the comparison between each treatment group and the PBS group are shown in Table S5.

In this liver fibrosis model, the mice had undergone five circRNAs injections over a 2-week period, providing an excellent condition for evaluating the safety and tolerability of the circRNAs therapeutics. We measured the serum levels of interferon (IFN)-α, tumor necrosis factor (TNF)-α, and interleukin (IL)-6 and found that both cRLN1 and cRLN2 treatments restored these cytokines to levels comparable to those of the Healthy group, indicating that the circRNA therapeutics exhibit excellent tolerability (Figure 2D).

To investigate whether relaxin inhibits hepatic fibrosis by interfering with the TGF-β1/Smad2 signaling pathway, we performed western blot analysis to examine the expression of TGF-β1, phosphorylated Smad2 ([pSmad2], the activated form of Smad2), α-smooth muscle actin ([α-SMA], a myofibroblast marker, encoded by *ACTA2*), and type I collagen ([Col1], a major ECM protein) in liver fibrosis model mice across different treatment groups.^53^ In liver fibrosis model mice, treatment with cRLN1 or cRLN2 markedly reduced TGF-β1 expression, subsequently decreased Smad2 phosphorylation, and ultimately lowered the expression and deposition of α-SMA, Col1, and hydroxyproline (reflecting total collagen content) (Figures 2E and 2F).^54^ In addition, the negative regulator of the TGF-β1/Smad2 pathway, cAMP, was significantly elevated in the serum of circRNA-treated groups compared to PBS and RLN1 protein treatments, with no significant difference between the two circRNAs (Figure 2G).^55^ In addition, immunohistochemistry (IHC) for α-SMA in liver tissues revealed a 3-fold reduction in the cRLN1 and cRLN2 treatment groups compared to PBS, while collagen expression also showed a 3-fold decrease as judged by Masson’s trichrome and sirius red staining (Figure 2H), further confirming reduced liver injury. Collectively, these results indicated that human relaxin-2 can exert similar biological functions as murine relaxin-1 in mice to effectively alleviate liver fibrosis.

### Reversing the cellular and molecular features in fibrotic livers with cRLN treatments

**Figure 3:**
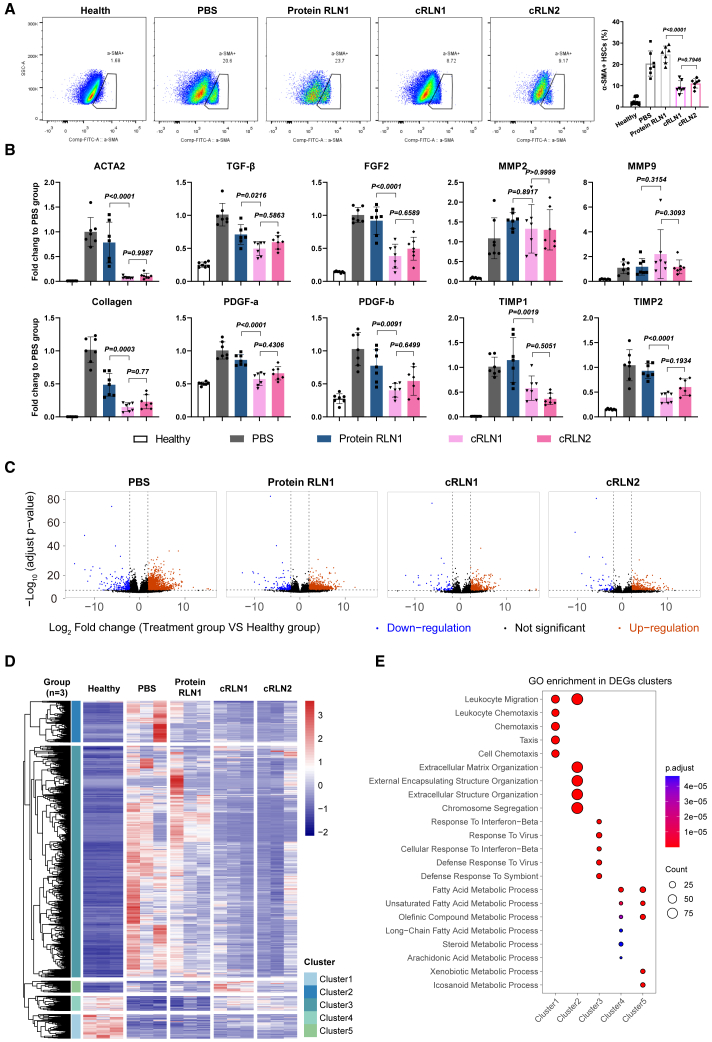
Structural and functional recovery of fibrotic livers after cRLN treatment (A) Flow cytometry analysis of α-SMA+ HSCs, quantifying HSC activation (*n* = 7 mice). (B) Relative mRNA expression of *ACTA2*, *Collagen I*, *TGF-β*, *FGF2*, *PDGF-a/b*, *MMP2/9*, and *TIMP1/2* in fibrotic livers across treatment groups (*n* = 7 mice). (C) Volcano plots of DEGs between treatment groups and Healthy controls (red: upregulated; blue: downregulated; *n* = 3 mice). (D) Heatmap of DEGs across treatment groups and Healthy controls (*n* = 3 mice). (E) GO enrichment analysis for biological process in five DEGs clusters derived from (C). Dot color indicates adjusted *p* value; dot size reflects DEG number. Experiments were repeated twice independently with similar results. Significant differences were assessed using a one-way ANOVA with Tukey test (A and B). Results are presented as mean ± SD from the second repeat. The *p* values for the comparison between each treatment group and the PBS group are shown in Table S5.

The fibrotic liver undergoes complicated structural and functional changes, including activation of HSCs, proliferation and fibrogenesis of the activated HSCs, and deposition of ECM. All these changes lead to liver damage characterized by vascular and biliary structural alterations, as well as impaired metabolic and detoxification functions.^4^ To determine the activation of HSCs, we used flow cytometry to measure the level of α-SMA^+^ cells, a major marker for activated HSCs.^25^ Our results showed that cRLN1 and cRLN2 treatment reduced HSC activation by about 2.2- and 1.8-fold, respectively (Figures 3A and S3). As expected, we observed the downregulation of *Col1* and *ACTA2* associated with reduced HSC proliferation and fibrogenesis after cRLN1 and cRLN2 treatments (Figure 3B). In addition, the expression of pro-fibrogenic factors, such as *TGF-β*, fibroblast growth factor 2 (*FGF2*), and platelet-derived growth factors (*PDGFs*), was significantly decreased by the treatments (Figure 3B). Notably, we observed the upregulation of *MMPs* and downregulation of tissue inhibitors of metalloproteinases (*TIMPs*), indicating the repair of fibrotic liver through degradation of collagen deposit (Figure 3B).

We further examined transcriptomic changes in mouse livers across treatment groups by sequencing total mRNA (*n* = 3 per group). Principal-component (PC) analysis of global transcriptome profiles revealed distinct clustering patterns (Figure S4A). Along PC1 (accounting for >60% of variance), the transcriptomes of Healthy and PBS-treated groups were widely separated, whereas cRLN1 and cRLN2 groups clustered closer to Healthy controls, suggesting partial restoration of gene expression profiles. We then identified differentially expressed genes (DEGs) in fibrotic livers (Figure 3C) and clustered significantly up- and downregulated genes (Figures 3D and S4B). The hierarchical clustering of these DEGs demonstrated that cRLN1 and cRLN2 treatment largely restored the gene expression patterns toward those of healthy mice (Figure 3D), particularly in the two largest clusters (cluster 1 and 2). Subsequent Gene Ontology (GO) analysis of these clusters revealed the significant downregulation of pathways related to chemotaxis, ECM organization, and cell mitosis in the cRLN1 and cRLN2 treatment groups compared to the PBS controls (Figure 3E). Furthermore, genes enriched in many metabolic pathways were upregulated in the cRLN1 and cRLN2 treatment groups (Figures 3D and 3E) toward the levels of Healthy controls, suggesting partially restored liver metabolic functions by the treatments (Figure 3E).

### Comparison of relaxin-2 expression from unmodified circRNAs and modified linear mRNA

**Figure 4:**
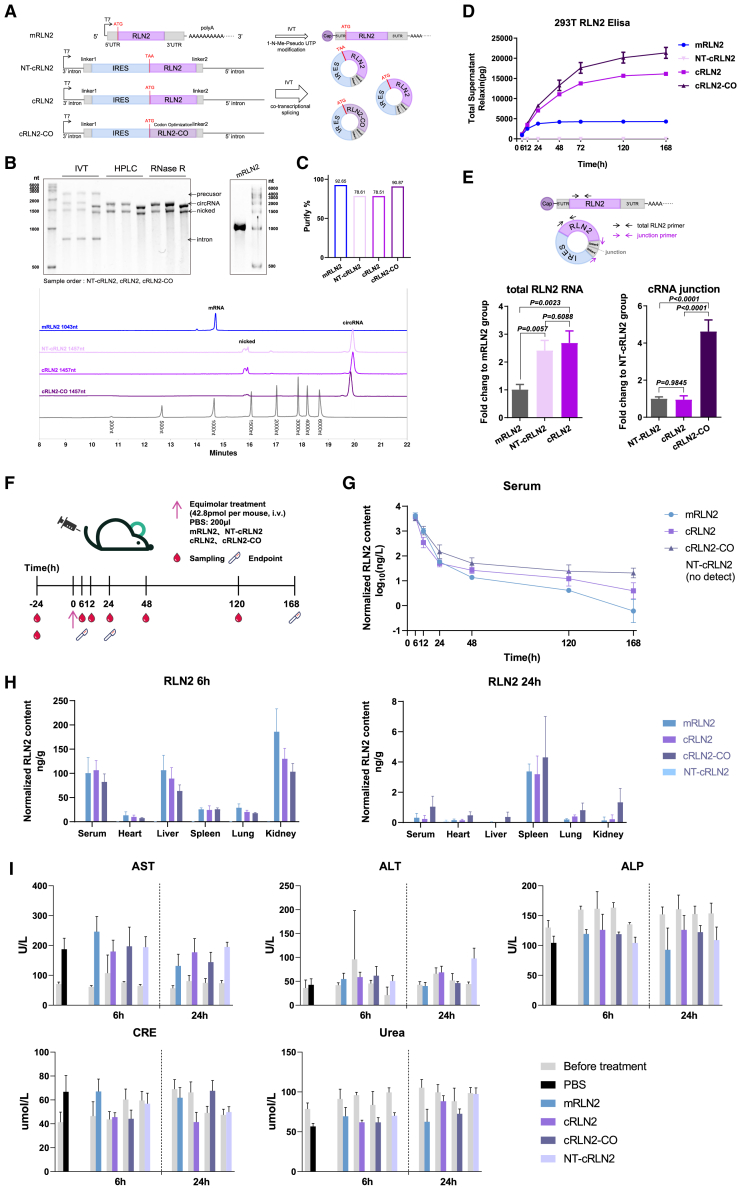
Pharmacokinetics and biosafety comparison between modified mRNA and circRNAs (A) Structures of mRNA and circRNAs encoding RLN2. mRLN2 contains native human 5′/3′ UTRs and CDS with *N*^1^Ψ modification, m^7^G cap, and 100 nt poly(A) tail. Three circRNAs share the same circularization system: NT-cRLN2 (ATG→TAA mutation, native CDS), cRLN2 (native CDS), and cRLN2-CO (codon-optimized CDS). (B) 4M-1.5% urea-denaturing agarose gel electrophoresis of NT-cRLN2, cRLN2, and cRLN2-CO before and after purification. Pre-purification circRNAs show four major stripes: precursor (2,313 nts), circRNAs (>1,457 nts), nicked circRNAs (1,457 nts), and intron (856 nts). Post-purification circRNAs display circRNAs and nicked circRNA stripes. Post-purification mRLN2 displays a major stripe (1,043 nts). (C) PA800 purity analysis of mRNA and circRNAs using Thermo RNA Ladder 6000. Main peak percentages quantify purification rates for mRLN2 (1,043 nts), NT-cRLN2, cRLN2, and cRLN2-CO (all 1,457 nts). Quantification of main peak of PA800 to reflect the purification of RNAs. (D) RLN2 expression kinetics in 293T cells transfected with equal RNA masses. Media RLN2 concentrations were measured at different time points to calculate cumulative production (*n* = 3 biological replicates). (E) Residual exogenous RNA in 293T cells at 168 h post-transfection. qPCR was performed using RLN2 CDS primers (for mRLN2/NT-cRLN2/cRLN2) or junction primers (for NT-cRLN2/cRLN2/cRLN2-CO), normalized to controls (*n* = 3 biological replicates). (F) Pharmacokinetic study design in healthy mice. After i.v. injection of different drugs (42.8 pmol mRLN2, NT-cRLN2, cRLN2, or cRLN2-CO), the blood and tissue samples were collected at different time points (*n* = 3 mice). (G) Serum RLN2 levels over a span of 168 h post-injection (*n* = 3 mice). In the order of the mRLN2, cRLN2, and cRLN2-CO treatment groups, the Cmax values were 4,246.43, 3,796.83, and 3,212.36, respectively; the AUC_0–168 h_ values were 158.4, 211.9, and 273.5 log_10_(ng/L)·h, respectively; and the t½ values were 20.52, 11.04, and 14.15 h, respectively. (H) RLN2 biodistribution in major organs at 6/24 h post-injection (*n* = 3 mice). (I) Serum ALT, AST, ALP, CRE, and urea levels at baseline, 6 h, and 24 h post-injection, evaluating hepatorenal toxicity (*n* = 3 mice). For all panels, both *in vitro* and *in vivo* experiments were repeated twice independently with similar results. Significant differences were assessed using a one-way ANOVA with Tukey test (E). Results are presented as mean ± SD in (D, E, G–I).

Compared to unmodified mRNA, unmodified circRNAs possess greater stability and enable a longer-lasting therapeutic effect, making them more suitable for chronic diseases like liver fibrosis.^56^ In addition, the purified circRNAs have a low tendency to induce innate immune responses even without base modifications.^57^^,^^58^ However, a direct comparison between the modified mRNA and unmodified circRNA encoding the same therapeutic protein is lacking. To rigorously evaluate circRNAs as next generation of mRNA therapeutics, we compared cRLN2 with *in vitro*-synthesized mRLN2 (containing a 5′ cap, poly-A tail length of 100 nts, and complete base modifications with *N*^1^-methylpseudouridine [*N*^1^Ψ] substitution on all uridines).^59^ As an additional control, we also mutated the start codon of cRLN2 into a stop codon (TAA), generating a non-translatable circRNA (NT-cRLN2) to assess any possible intrinsic anti-fibrotic effects by non-coding circRNAs. To enhance protein expression efficiency from circRNAs, we also optimized the ORF of cRLN2 to generate cRLN2-CO (Figure 4A).

All circRNAs were synthesized *in vitro* and purified with HPLC (Figure S5A). After HPLC purification, the circRNAs showed a single stripe on a 1% low-melting-point agarose gel, but two stripes corresponding to circRNAs and nicked circRNAs were observed on a urea-denaturing agarose gel (Figures 4B and S5C). Nicked circRNAs were digested with RNase R, and PA800 analysis showed that the circularization efficiency of the circRNAs reached approximately 80% (Figure 4C). The synthesis and purification of mRLN2 were outsourced to Suzhou Novoprotein Biotechnology Co., Ltd (Figure S5B). The purified mRLN2 showed a single stripe on a 1% low-melting-point agarose gel and urea-denaturing agarose gel (Figures 4B and S5C). The purity of mRLN2 reached 92.65% as determined by PA800 analysis (Figure 4C).

Equal masses of the four RNAs were transfected into 293T cells, and RLN2 secretion was monitored over 168 h with periodic medium replacement (Figure 4D). As expected, NT-cRLN2 did not produce any detectable RLN2, whereas cRLN2 mediated robust RLN2 expression with cumulative levels 3.8-fold higher than mRLN2 in the time window of 0–168 h (Figure 4D). In comparison, the mRLN2 showed a lower level of RLN2 expression that was undetectable after 72 h (Figure 4D). In addition, the codon optimization further increased protein expression from cRLN2-CO, with cumulative protein level at ∼1.3-fold higher than that from cRLN2 after 168 h post-transfection (Figure 4D). The quantitative reverse-transcription PCR (RT-qPCR) experiments at 168 h post-transfection revealed that the RNA levels of NT-cRLN2 and cRLN2 were 2.4- and 2.7-fold higher than that of mRLN2, respectively (Figure 4E), indicating higher stability of circRNAs *in vitro*. Measurement of the circRNAs by detecting the junction sites showed that the cRLN2-CO levels were 4.6-fold higher than the other two circRNAs (Figure 4E), suggesting that codon optimization further improves circRNAs stability *in vitro*.

Next, we encapsulated all four RNAs in ALC-0315 LNPs, achieving encapsulation efficiencies above 80%, an average size ranging from 75 to 100 nm, a polydispersity index below 0.4, and a zeta potential of approximately ±15 mV (Table S1). These results indicate the successful production of high-quality reporter LNPs. After i.v. injection of equimolar doses of RNAs into healthy mice (Figures 4F and S6A), we found that the serum relaxin peaked at 6 h for both mRLN2 and cRLN2 but declined significantly by 24 h (Figure 4G). Interestingly, cRLN2 experienced a more pronounced decrease of protein production at 12 h, with expression dropping two orders of magnitude from peak levels. Codon optimization effectively mitigated the rapid decline of cRLN2 within the first 24 h *in vivo*, resulting in higher expression and longer expression time. The peak plasma concentrations (Cmax) of mRLN2, cRLN2, and cRLN2-CO were 4,246.43, 3,796.83, and 3,212.36 ng/L, respectively, and the terminal elimination half-lives (t½) were 20.52, 11.04, and 14.15 h, respectively. These two parameters indicate that mRNA has an *in vivo* advantage 24 h after injection (Figure 4G). Subsequently, cRLN2 and cRLN2-CO maintained a slowly declined low-level RLN2 expression until 168 h post-injection, while mRLN2 declined more rapidly and fell below detection limits at approximately 120 h post-injection. The areas under the concentration-time curve (AUC_0–168 h_) of mRLN2, cRLN2, and cRLN2-CO were 158.4, 211.9, and 273.5 log_10_(ng/L)·h, respectively. This indicates that the expression of circRNAs remains at a higher level than that of mRNA between 24 and 168 h post-injection (Figure 4G). A repeat experiment on the serum level of relaxin after the injection of cRLN2 and mRLN2 confirmed these trends (Figure S6B).

At peak expression (6 h), RLN2 was predominantly found in the liver and kidneys, with serum levels comparable to hepatic levels (Figure 4H). By 24 h post-injection, the relaxin expression from exogenous RNA had significantly declined and the RLN2 protein was primarily detected in the spleen with minimal levels in the liver (Figure 4H). In the repeat experiments, we collected liver samples at different time points from three mice to measure relaxin protein levels and again found that protein expression from mRLN2 and cRLN2 decline rapidly in the liver (Figure S6C). The RT-qPCR experiment showed comparable low levels of RNAs in the liver at 72 h for both mRLN2 and cRLN2 (Figure S6D). Interestingly, cRLN2-CO exhibited the slowest decline in protein expression, demonstrating greater stability and more persistent translation both in serum and different tissues (Figures 4G and 4H).

Importantly, neither modified mRNA nor unmodified circRNAs induced liver damage responses in healthy mice, as evidenced by similar AST and ALT levels between the treatment groups and the PBS-treated mouse from 6 to 168 h post-injection (Figures 4I and S6E). The levels of alkaline phosphatase (ALP), creatinine (CRE), and urea also remained normal after the injection (Figures 4I and S6E), indicating that the circRNAs or modified mRNAs do not stimulate hepatic or renal inflammation from 6 to 168 h post-injection. Furthermore, late histopathology (168 h) showed no significant morphological differences in the heart, liver, spleen, lung, or kidney between RNA-treated and PBS groups, confirming the biosafety of circRNAs as nucleic acid drugs (Figure S6F).

### Unmodified cRLN2 exhibited comparable therapeutic efficacy to modified mRLN2 in the liver fibrosis model

In the liver fibrosis mouse model, we validated that cRLN2 at a dose of 42.8 pmol per mouse significantly reversed liver fibrosis. Pharmacokinetic studies revealed that cRLN2 undergoes faster initial suppression of expression than mRLN2 upon entering the body but maintains a long-term low-level expression (Figure 4G). To compare the therapeutic efficacy of cRLN2 and modified mRLN2 in the liver fibrosis model and determine the optimal cRLN2 dose, we tested three cRLN2 dose groups: 10.7 pmol (low dose), 42.8 pmol (medium dose), and 85.6 pmol (high dose), with corresponding low and medium doses of modified mRLN2 as controls.

**Figure 5:**
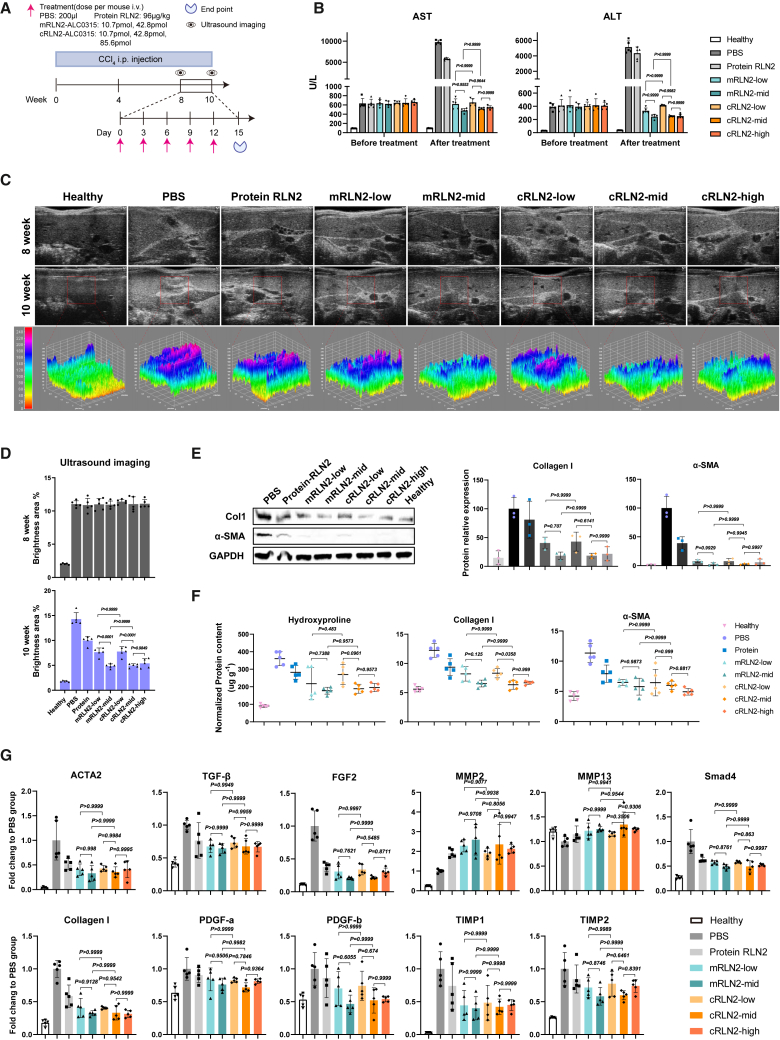
cRLN2 exhibited comparable anti-fibrotic efficacy to equimolar mRLN2 (A) CCl_4_-induced fibrosis model with treatment groups: PBS, protein RLN2 (96 μg/kg), mRLN2-ALC0315 (10.7/42.8 pmol), and cRLN2-ALC0315 (10.7/42.8/85.6 pmol). (B) Hepatic injury reflected by serum ALT and AST levels at weeks 8 (before treatment) and 10 (after treatment), *n* = 5 mice. (C) Representative ultrasound images at weeks 8 and 10, with 3D surface plots (week 10) showing echotexture. (D) Fibrosis severity was quantified by integrated echointensity in the brightness area from the imaging of (C) (*n* = 5 mice). (E) Western blot analysis of α-SMA/collagen I using the liver lysis (representative of three experiments; ImageJ quantification; *n* = 3 mice). (F) Quantification of ELISA results for hydroxyproline, collagen I, and α-SMA in liver tissue (*n* = 5 mice). (G) Relative mRNA expression of *ACTA2*, *Collagen I*, *TGF-β*, *FGF2*, *PDGF-a/b*, *MMP2/9*, *TIMP1/2*, and *Smad4* in fibrotic livers across treatment groups (*n* = 5 mice). Experiments were repeated twice independently with similar results. Significant differences were assessed using a one-way ANOVA with Tukey test (B, D–G). Results are presented as mean ± SD from the second repeat. The *p* values for the comparison between each treatment group and the PBS group are shown in Table S5.

We induced liver fibrosis in 8-week-old male mice via intraperitoneal injections of CCl_4_, and monitored the fibrosis progression using liver ultrasonography (Figure 5A). By week 8, AST and ALT levels were significantly elevated (Figure 5B), and liver echogenicity indicated severe fibrosis of the animals (Figures 5C and 5D). We subsequently administered the different RNA drugs via i.v. injection at varying doses, with high dose of human relaxin-2 protein as a control. Compared to PBS control, modified mRLN2 and cRLN2 treatment groups showed significantly reduced liver echogenicity and partial reversal of elevated ALT and AST levels. The *p* values for the comparison between each treatment group and the PBS group are shown in Table S5. In addition, we observed significantly decreased hydroxyproline, collagen I, and α-SMA expression after RNA drug treatments (Figures 5E and 5F), suggesting a reversal of liver fibrosis. Both modified mRNAs and circRNAs showed better efficacy than the group treated with RLN2 protein even in the lowest doses, probably due to the short half-life of relaxin protein. We also examined the expression of the biomarkers of fibrosis and confirmed the downregulation of fibrogenic genes (*TGF-β*, *FGF2*, *PDGFs*, *TIMPs*, and *Smad*) and the upregulation of anti-fibrotic genes (*MMPs*) (Figure 5G). The morphological examination of liver revealed severely damaged and distorted structures in PBS-treated mice, while the treated groups exhibited dose-dependent restoration toward healthy morphology (Figure S7A). Moreover, histological staining with α-SMA, sirius red, and Masson’s trichrome all showed reduction of liver fibrosis in the RNA treatment groups (Figures S7A and S7B). As expected, we also observed similar reduction of activated HSCs in all the RNA-treated groups (Figure S7C). Equimolar doses of modified mRLN2 and cRLN2 showed no significant differences in any of the parameters related to the alleviation of liver fibrosis, regardless of whether low or medium doses were administered.

Compared with the PBS group, even low-dose cRLN2 treatment produced a significant therapeutic effect. However, for certain parameters, such as ultrasound imaging metrics, collagen quantification, and histological scoring, there were significant differences between low- and medium-dose cRLN2 treatments, indicating that medium-dose cRLN2 is more effective in alleviating liver fibrosis than low-dose treatment (Figures 5D, 5F, and S7B). In addition, high-dose cRLN2 showed no significant differences from medium-dose cRLN2 across all measured parameters, suggesting that medium-dose cRLN2 represents the optimal dosing regimen. These results also suggested that cRLN2 treatment had a broad therapeutic window, as we did not observe any adverse effect in the high-dose group.

### The cRLN2-CO did not significantly improve therapeutic outcomes under current dosing regimens

**Figure 6:**
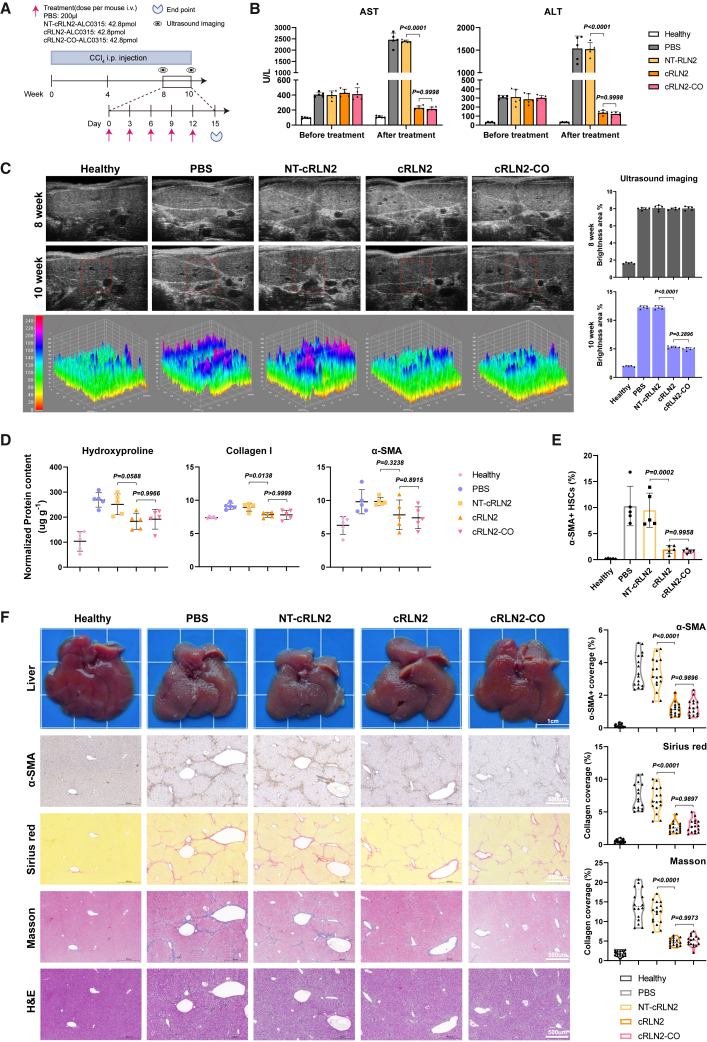
Codon-optimized cRLN2 showed no significant therapeutic improvement under current dosing regimen (A) CCl_4_-induced fibrosis model with treatment groups: PBS, NT-cRLN2, cRLN2, and cRLN2-CO (42.8 pmol). (B) Serum ALT and AST levels at weeks 8 (before treatment) and 10 (after treatment), *n* = 5 mice. (C) Representative ultrasound images at weeks 8 and 10, with 3D surface plots of week 10 samples showing echotexture. Fibrosis severity was quantified by integrated echointensity in the bright area (*n* = 5 mice). (D) Quantification of ELISA results for hydroxyproline, collagen I, and α-SMA in liver tissue (*n* = 5 mice). (E) Flow cytometry analysis of α-SMA+ HSCs (*n* = 5 mice). (F) Liver morphology and histology (IHC: α-SMA, sirius red, Masson’s trichrome, H&E). Scale bars: 1 cm for liver images and 500 μm for histology sections. Quantification from four random fields per mouse (*n* = 4 mice). Experiments were repeated twice independently with similar results. Significant differences were assessed using a one-way ANOVA with Tukey test (B–F). Results are presented as mean ± SD from the second repeat. The *p* values for the comparison between each treatment group and the PBS group are shown in Table S5.

Our pharmacokinetic results showed that, compared to cRLN2, the serum relaxin-2 produced from cRLN2-CO avoided the rapid decline at 12 h and maintained higher expression levels after 24 h upon systemic delivery (Figure 4G). Therefore, we next examined if the cRLN2-CO may exhibit superior efficacy over cRLN2 in reversing liver fibrosis at the optimal dose (42.8 pmol, medium dose). To this end, we conducted a direct comparison of cRLN2-CO with cRLN2 using the same liver fibrosis model (Figure 6A). Before the treatment (week 8), we confirmed that the serum AST and ALT levels were significantly elevated (Figure 6B) with hyperechoic liver texture presented (Figure 6C), suggesting severe fibrosis. The mice were then treated for 2 weeks with i.v. injections of equimolar cRLN2 or cRLN2-CO (42.8 pmol), using parallel treatment by NT-cRLN2 and PBS as negative controls.

Both cRLN2 and cRLN2-CO treatments significantly decreased AST and ALT levels (Figure 6B) and liver echogenicity (Figure 6C), indicating a reversal of liver fibrosis. Consistently, the treatments caused significant decreases in several fibrosis markers (hydroxyproline, collagen I, and α-SMA) (Figures 6D and S8B), and reduction of activated HSCs (Figures 6E and S8A). In contrast, NT-cRLN2 showed no therapeutic effect, confirming that the anti-fibrotic activity depended on relaxin-2 translation rather than circRNA itself. Furthermore, both cRLN2 and cRLN2-CO showed comparable efficacy in downregulating profibrotic genes (*Smad*, *TGF-β*, *FGF2*, *PDGFs*, and *TIMPs*) and upregulating antifibrotic *MMPs* (Figure S8C). Consistent with phenotypic rescue, both cRLN2 and cRLN2-CO treatments restored liver morphological architecture (Figure 6F), supported by reduced fibrosis in α-SMA, sirius red, and Masson’s trichrome staining (Figure 6F). Interestingly, similar therapeutic efficacies were observed from both cRLN2 and cRLN2-CO (Figures 6C–6F), despite cRLN2-CO producing a higher and more prolonged expression of relaxin protein in cultured cells (Figure 4D) and mice (Figure 4G), probably due to the sufficient levels of protein production from both circRNAs at the condition we tested.

## Discussion

CircRNA has emerged as a next-generation of mRNA therapeutics, offering enhanced stability and reduced immunogenicity.^60^ In this study, we demonstrate that circRNAs encoding human relaxin-2 represent a new and effective therapeutic strategy for liver fibrosis. We systematically compared the pharmacokinetics, biosafety, and therapeutic effects of several unmodified circRNAs and modified mRNAs in both healthy mice and liver fibrosis models and further conducted dose-escalation experiments to determine the optimal dosing regimens. Our findings reveal several key advances in the development of circRNA-based therapeutics: (1) successful engineering of circRNAs capable of robust relaxin-2 production through IRES-mediated translation, (2) superior pharmacokinetic properties compared to conventional mRNA therapies, and (3) substantial anti-fibrotic efficacy with an excellent safety profile in mouse models.

Notably, comparable anti-fibrotic efficacy was found between human and murine relaxin-encoding circRNAs, suggesting the cross-species activity of RLN2 in mouse models. This finding validated the use of mouse models for preclinical development of human RLN2 therapies and implied that species-specific optimization may not be necessary for clinical translation.

The safety profile of our circRNAs constructs was particularly encouraging. In the pharmacokinetic study conducted in healthy mice, animals were sacrificed at 6, 24, 48, 72, 120, and 168 h after a single injection of the cRLN2 (Figure S6A). Blood biochemical parameters showed no significant differences compared with the PBS group, indicating that circRNA administration does not impair liver, biliary, or kidney function (Figure S6E). Furthermore, late-stage (168 h) histopathological analysis of the heart, liver, spleen, lungs, and kidneys revealed no notable tissue alterations, suggesting that circRNAs exert minimal impact on major organs *in vivo* (Figure S6F). In a severe liver fibrosis model, treatment with cRLN2 not only effectively alleviated fibrosis but also restored the normal levels of inflammatory cytokine in serum (IFN-α, TNF-α, and IL-6) (Figure 2D). Notably, the levels of inflammatory cytokines were comparable to those of the healthy group even after five injections in a span of 2 weeks, suggesting a good safety profile for such treatment. Furthermore, despite the known immunostimulatory potential of exogenous RNA,^61^ we observed no significant inflammatory responses or organ toxicity even at high doses (Figures 6 and S7). This safety profile, combined with the ability to achieve therapeutic effects at relatively low doses (10.7–42.8 pmol), suggests a favorable risk-benefit ratio for clinical development. Collectively, these data confirm the outstanding safety and excellent tolerability of circRNAs, establishing a foundation for their development as a next-generation mRNA therapeutic platform.

A systematic comparison between circRNAs and modified mRNA platforms reveals several notable differences. In the cellular experiments, circRNAs exhibit higher expression levels and greater stability than modified mRNAs. In healthy mice, circRNAs performed less favorably than mRNAs during the first 24 h after injection, with both lower Cmax and shorter t½. However, beyond 24 h, circRNAs showed a prolonged and stable expression, resulting in a higher AUC_0–168 h_ compared with modified mRNAs. This result implies that circRNAs may be better suited for therapeutic applications requiring long-term, sustained expression. Interestingly, circRNAs displayed a transient decrease in expression in the liver before stabilizing at a lower level, a pattern that may result from rapid degradation triggered by an immunological response to high circRNA doses. Elucidating the underlying mechanisms of this response will be crucial for advancing circRNA-based therapeutics. Unlike mRNAs that require extensive chemical modification to reduce immunogenicity, unmodified circRNAs maintain low innate immunogenicity, while achieving therapeutic efficacy comparable to that of modified mRLN2 in the liver fibrosis model.

As an emerging RNA therapeutic platform, circRNA-based drugs may be benefited from further optimizations through several directions.

(1) Certain RNA modifications. A recent study revealed that when LNP-delivered mRNA disrupts endosomal membranes, the released protons (H^+^) activate TRIM25, thereby promoting RNA degradation.^62^ However, incorporating *N*^1^Ψ into mRNA significantly reduces TRIM25 binding. However, it is worth noting that *N*^1^Ψ may also induce +1 ribosomal frameshifting and cellular immune responses,^63^ which is a potential advantage of circRNAs over mRNAs as a new therapeutic platform because they do not require *N*^1^Ψ modification. Studies have shown that nicked circRNAs may also exhibit potential immunogenicity, which can be reduced by pseudouridine modification.^64^ In addition, introducing a cap structure and a poly(A) sequence into circRNAs through chemical modification can further enhance their translational efficiency.^65^^,^^66^
(2) Sequence optimization. Systematic optimization of IRES elements, m^6^A motifs, UTRs, CDS regions, RNA length, and structural features can improve the translational efficiency of circRNAs. In this study, codon optimization of the CDS (cRLN2-CO) led to higher translation efficiency *in vitro* compared with the non-optimized cRLN2. In healthy mice, cRLN2-CO exhibited a lower Cmax than cRLN2 but showed longer t½ and a higher AUC_0–168 h_, indicating a more stable pharmacokinetic profile. However, cRLN2-CO demonstrated similar therapeutic efficacy to cRLN2 at equivalent doses in the liver fibrosis model, likely due to dose saturation. Given that the PK advantage of cRLN2-CO lies in its high, sustained expression during later time points, the current 2-week dosing regimen—one injection in every 2 days—may not adequately reveal the benefits of codon optimization. Future studies should explore extended dosing intervals, reduced dosing frequency, and lower dose levels.
(3) RNA delivery. Current LNP-mediated delivery systems are optimized primarily for mRNA vaccines that require transient, high-level antigen expression and thus are not yet tailored for therapeutics requiring long-term, sustained expression. In our pharmacokinetic study, the target protein was primarily detected in the liver and kidneys during the early phase, whereas in the later phase, it was predominantly detected in the spleen. We speculate that such distribution pattern may be related to the properties of the LNP formulation. The ALC-0315 LNP primarily mediates hepatocyte uptake through ApoE-dependent mechanisms, but particles larger than 80 nm or with excessive polyethylene glycol (PEG) modification may be sequestered by the spleen or lung, reducing liver targeting and resulting in predominant protein expression in the spleen.^46^ In the future, LNP-based delivery studies for circRNA in protein replacement therapy should focus on optimizing physicochemical properties, enhancing tissue-targeted delivery, and achieving sustained release to better support chronic treatment.

Several limitations of our study warrant further discussion. First, although both cRLN2 and mRLN2 treatments effectively alleviated liver fibrosis, they did not fully restore the mice to a healthy state. This is partly because the mice continued to receive CCl_4_ injections during the treatment period and partly because the fibrosis model was excessively severe. Most studies using relaxin to treat liver fibrosis employ a mild fibrosis model induced for 1 month, whereas our model involved 2 months of induction, resulting in severe fibrosis. We chose a severe fibrosis model mainly because the mild liver fibrosis may be managed by dietary intervention and lifestyle changes and the future patients who likely gain the most benefits from RNA-based therapy will be those with severe fibrosis. Second, our evaluation was limited to a 2-week treatment period; longer-term studies will be needed to assess durability of effect and potential late-onset toxicity.^67^^,^^68^ Finally, while ALC-0315-based LNP provided excellent liver targeting in mice, this formulation was originally developed for single-dose rapid release in COVID-19 vaccines. The optimal LNP formulations for sustained circRNAs delivery in chronic diseases remain to be developed.

Taken together, our findings position circRNA as a robust therapeutic platform for chronic fibrotic diseases, characterized by its prolonged durability, excellent safety profile, and potent anti-fibrotic efficacy. To accelerate clinical translation, future research should focus on scaling productions, optimizing dosing regimens, and evaluating efficacy in additional preclinical models to facilitate translation to human trials.

## Materials and methods

### Plasmid design

All plasmid vectors used for the preparation of circRNA were derived from a flexible, efficient, and scalable platform previously developed by our research group.^51^ The platform employs homologous recombination to clone the target fragment expressing relaxin into a backbone containing the T7 RNA polymerase promoter and terminator, a group II intron from *Clostridium tetani*, and the NheI and XbaI restriction sites from CVB3. The coding sequences (CDSs) of the circRNAs used in this study are listed in Table S3.

### circRNA preparation and validation

Plasmid DNA was linearized by digestion with XbaI and purified using a phenol-chloroform-isoamyl alcohol mixture (Sigma). Linearized DNA was subjected to IVT in the presence of unmodified NTPs using the T7 RiboMAX Express Large-Scale RNA Production System (Promega) to synthesize cRLN1, cRLN2, NT-cRLN2, and cRLN2-CO. After DNase I treatment, the RNA products were purified using lithium chloride (Thermo Fisher) to remove excess NTPs and salts from the IVT buffer. The RNA was further purified by HPLC (Shimadzu) using a size-exclusion chromatography column (XBridge Protein BEH SEC Column, 450 Å, 3.5 μm, 7.8 mm × 300 mm). The mobile phase consisted of 10 mM Tris, 1 mM EDTA, and 75 mM PB (pH 7.4) at 25°C, with a flow rate of 0.5 mL/min. Fractions were collected and confirmed by 1% agarose gel electrophoresis. After HPLC purification, circRNAs with a single peak were reverse transcribed into cDNA using ABScript *Neo* RT Master Mix for qPCR with gDNA Remover (ABclonal). The junction sites were verified by Sanger sequencing, with primers listed in Table S2. These circRNAs were treated with RNase R (LGC) to further eliminate nicked circRNAs, followed by column purification (ZYMO) and monitoring with a PA800 system to obtain circRNAs with optimal cyclization efficiency. Biochemical characterization of circRNAs was performed using a 1.5% vertical agarose gel containing 4 M urea, with TBE as the running buffer. Electrophoresis was carried out at a constant power of 15 W at room temperature for approximately 15–20 min.

### mRNA preparation

mRLN2 was synthesized via T7 polymerase-mediated IVT from a linearized pCMV plasmid vector. The transcription reaction included CleanCap AG (Cap1) and *N*^1^Ψ. The poly(A) tail was co-transcriptionally added in a template-dependent manner. Uridine-5′-triphosphate (UTP) was completely replaced with *N*^1^Ψ to enhance protein translation and reduce the immunogenicity of synthetic mRNA. The IVT reaction was outsourced to Suzhou Novoprotein Biotechnology Co., Ltd. The purity of the synthesized mRNA was verified by gel electrophoresis and PA800. The CDSs of the mRNAs used in this study are listed in Table S3.

### Cell culture

293T cells (purchased from the Cell Bank of the Chinese Academy of Sciences, SCSP-502) were cultured in DMEM (Gibco) supplemented with 10% fetal bovine serum ([FBS], Procell, China) at 37°C in a humidified atmosphere containing 5% CO_2_.

### *In vitro* transfection

293T cells were seeded at a density of 2 × 10^5^ cells per well in white, clear-bottom 12-well plates (Thermo Fisher). On the next day, cells were transfected with 1 μg of pRLN1 using Lipofectamine 3000 (Thermo Fisher) or with cRLN1 and cRLN2 using Lipofectamine MessengerMAX (Thermo Fisher). The concentration of relaxin in the cell culture medium was measured before transfection and at 6, 12, 24, and 48 h post-transfection using the Human Relaxin-2 Quantikine ELISA Kit (R&D Systems) and Mouse Relaxin 1 ELISA Kit (Boster Bio).

### *In vitro* pharmacokinetics

293T cells were seeded at 5 × 10^4^ cells per well in white, clear-bottom 12-well plates (Thermo Fisher). The following day, cells were transfected with 1 μg of mRLN2, NT-cRLN2, cRLN2, or cRLN2-CO using Lipofectamine MessengerMAX (Thermo Fisher). The culture medium was replaced at 6, 12, 24, 48, 72, 120, and 168 h post-transfection, and the concentration of RLN2 in the medium was measured using the Human Relaxin-2 Quantikine ELISA Kit (R&D Systems). The relative amount of exogenous RNA was quantified by qPCR using total RNA from cells at 168 h post-transfection. Total RNA was extracted with TRIzol (Thermo Fisher), reverse transcribed with ABScript *Neo* RT Master Mix for qPCR with gDNA Remover (ABclonal), and amplified with 2X Universal SYBR Green Fast qPCR Mix (ABclonal). Primer sequences are listed in Table S2.

### LNP preparation and characterization

The mRNA and circRNAs were encapsulated in an LNP via the NanoAssemblr Ignite nanoparticle formulation systems (Cytiva). In brief, an aqueous solution of mRNA and circRNAs at pH 4.0 is rapidly mixed with a lipid mixture dissolved in ethanol, which contains different ionizable cationic lipid, distearoylphosphatidylcholine (DSPC), DMG-PEG2000, and cholesterol. The ratios for the lipid mixture are ALC-0315:DSPC:cholesterol:ALC-0159 = 46.3:9.4:42.7:1.6. The resulting LNP mixture was then dialyzed against 10% sucrose solution and stored at −80°C for further application (up to 12 months). The size and surface charge of the nanoparticles were determined by a ZetaPALS/BI-200SM (Brookhaven, USA) or Zetasizer Pro (Malvern Panalytical., USA). The encapsulation efficiencies are measured by RiboGreen RNA kit (ThermoFisher Scientific). To monitor the *in vivo* expression of cFluc-LNP, 8-week-old healthy male mice were i.v. injected with 20 μg of cFLuc-ALC0315 with PBS as a control. At 12 and 24 h post-injection, mice were intraperitoneally administered firefly luciferin (Yeasen Biotechnology) and imaged using an IVIS Lumina system (PerkinElmer).

### Mouse models

Eight-week-old male C57BL/6 mice were purchased from Shanghai SLAC Laboratory Animal Co., Ltd. and housed in a specific pathogen-free barrier facility (Room 4140, No. 3577 Jinke Road, Shanghai Laboratory Animal Research Center). Environmental conditions were maintained at 20°C–26°C, 40%–70% humidity, and a 12-h light/dark cycle. Mice were housed in individually ventilated cages (IVC; 320 × 220 × 135 mm) with sterilized corncob bedding, replaced weekly. Irradiated feed was provided by Jiangsu Synergetic Medical Bioengineering Co., Ltd., and autoclaved water was available ad libitum. After a 4-day acclimation period, mice were ear-tagged and randomly assigned to experimental groups. For the CCl_4_-induced liver fibrosis model, mice were intraperitoneally injected with a CCl_4_/corn oil mixture (4:1, v/v) at a dose of 10 μL/g body weight (e.g., 200 μL for a 20 g mouse) twice weekly for 10 weeks. Treatments began at week 8 and continued for 2 weeks. Liver injury was assessed 48 h after CCl_4_ injection at weeks 8 and 10 via ultrasound imaging, as well as by the measurement of serum AST and ALT levels. All procedures were approved by the Institutional Animal Care and Use Committee (IACUC) of the Shanghai Institute of Nutrition and Health, CAS (SINH-2023-WZF-1), and Southern University of Science and Technology (SUSTech-JY202402052-20240404A1).

### Pharmacokinetics and biosafety analysis in mouse

Eight-week-old healthy male mice were i.v. injected with 42.8 pmol of mRLN2-ALC0315, NT-ALC0315, cRLN2-ALC0315, or cRLN2-CO-ALC0315 with PBS as a control. Blood was collected 24 h before injection and at 6, 12, 24, 48, 120, and 168 h post-injection. Serum RLN2 levels were measured using the Human Relaxin-2 Quantikine ELISA Kit (R&D Systems). Additional cohorts were euthanized at 6 and 24 h for serum biochemistry and organ RLN2 analysis. In replicate experiments, mice were euthanized by cervical dislocation at 120 h, and organs (heart, liver, spleen, lung, and kidney) were harvested for histopathology (H&E staining).

### Efficacy studies

CCl_4_-treated mice were blindly randomized into treatment groups. Feasibility validation: PBS, cRLN1 (42.8 pmol, i.v.), cRLN2 (42.8 pmol, i.v.), protein RLN1 (32 μg/kg, i.v.). Dose escalation: PBS, cRLN2 (10.7, 42.8, 85.6 pmol, i.v.), mRLN2 (10.7, 42.8 pmol, i.v.), protein RLN2 (96 μg/kg, i.v.). Sequence impact: PBS, cRLN2 (42.8 pmol, i.v.), cRLN2-CO (42.8 pmol, i.v.). Liver fibrosis progression was monitored using a Vevo 2100 ultrasound system. Mice were euthanized 3 days after the final treatment for serum biochemistry, gross liver morphology assessment, immunohistochemistry, flow cytometry, ELISA, western blot, and qPCR.

### Liver ultrasound

Ultrasound imaging was performed using a Vevo 2100 high-frequency system (VisualSonics). Mice were anesthetized with 2.5% isoflurane, abdominal hair was removed with depilatory cream (Veet), and pre-warmed ultrasound gel (JUMPER) was applied. The liver was imaged in the parasternal long-axis view, with triplicate measurements. ImageJ 1.54 g was used to quantify echogenicity via integrated density.

### Immunohistochemistry and histological staining

Liver tissues were resected and rinsed in PBS and placed in 4% paraformaldehyde (PFA) overnight at 4°C. Following 4% PFA, tissues were rinsed in xylene and gradient ethanol concentrations until paraffin embedded. Immunohistochemistry and histological staining were performed on paraffin-embedded sections from fibrotic livers and other major organs. For immunohistochemistry analysis, tissue sections were boiled in sodium citrate buffer (10 mM, pH 6.0) for 30 min for antigen retrieval and treated with 3% hydrogen peroxide to inhibit endogenous peroxidase activity. The sections were treated with 5% goat serum to block nonspecific binding sites and incubated with primary anti-α-SMA antibody (1:1000, Boster) at 4°C overnight, followed by secondary HRP-conjugated goat anti-mouse IgG antibody (1:1,000, Abcam) at 37°C for 50 min. The color was developed using a DAB mixture (Solarbio). Masson’s trichrome staining was performed following the manufacturer’s protocols (Abcam). H&E and sirius red staining were following the manufacturer’s protocols (Shanghai Jingke Chemical Technology Co., Ltd., China, and Servicebio Ltd., China). Immunohistochemistry and histological images were taken by using a Digital Scanner (Pannoramic MIDI, 3DHISTECH Ltd., Hungary). The semi-quantification of fibrosis was conducted by measuring the average optical density of the histochemical or immunohistochemical reactant area using ImageJ 1.54 g software.

### Flow cytometry analysis

Liver cells were isolated from fibrotic mice through enzymatic digestion using a solution containing collagenase I (200 U/mL, Invitrogen), collagenase IV (200 U/mL, Invitrogen), and DNase I (100 μg/mL, Invitrogen) at 37°C for 30 min. The digestion was terminated by adding an equal volume of PBS supplemented with 10% FBS. The resulting cell suspension was filtered through a 40-μm cell strainer, followed by red blood cell lysis using RBC Lysis Buffer (BioLegend). For viability staining, 1 mL of the single-cell suspension was centrifuged and incubated with LIVE/DEAD Fixable Near-IR Dead Cell Stain (Thermo Fisher Scientific) at room temperature for 20 min in the dark. Cells were then stained with fluorescence-conjugated anti-mouse antibody cocktails (BioLegend) in 1× cell staining buffer (BioLegend) at 4°C for 20 min in the dark. The reaction was stopped by adding 1 mL of staining buffer, and 300 μL aliquot was prepared for analysis. Flow cytometry was performed on a BD FACS Canto system running FACSDiva 8.1 software (BD Biosciences). Viable cells were gated using Live/Dead Fixable Far Red Dead Cell Stain (Thermo Fisher Scientific), and data were analyzed with FlowJo 10.6 (Tree Star). Antibody specifications are provided in Table S4.

### Blood biochemical analysis

Collected mouse blood was allowed to clot at room temperature for 1 h, followed by centrifugation at 6,000 rpm for 15 min at 4°C to isolate serum. Serum levels of AST, ALT, ALP, CRE, and urea were measured using a fully automated biochemical analyzer (Ortho VITROS 4600, Ortho Clinical Diagnostics). Serum level of cAMP was measured using General Cyclic Adenosine Monophosphate ELISA Kit (cAMP) (Abclone RK09298). Serum levels of cytokines were measured using Mouse Interferon alpha-1 (Ifna1) ELISA Kit (Abclone RK06086), Mouse TNF-alpha ELISA Kit (Abclone RK00027), and Mouse IL-6 ELISA Kit (Abclone RK00008).

### Hydroxyproline assay

Liver hydroxyproline (HYP) content was determined using a Hydroxyproline Assay Kit (Solarbio) following the manufacturer’s instructions. Approximately 500 mg of tissue was minced in a glass tube and 5 mL of extraction buffer was added. The mixture was boiled until no visible tissue clumps remained. The pH was adjusted to 6–8 with 10 M NaOH (∼3 mL), and the volume was brought to 8 mL with distilled water. After centrifugation at 16,000 rpm for 20 min at 25°C, the supernatant was collected. A 200 μL aliquot of the supernatant was mixed with 200 μL of chloramine-T oxidation reagent and incubated at room temperature for 20 min. Then, 200 μL of *p*-dimethylaminobenzaldehyde developer was added, and the mixture was incubated at 60°C for 20 min, followed by 15 min at room temperature. Absorbance at 560 nm was measured, and HYP content was calculated by comparison with the standard curve.

### Protein quantification by ELISA

Approximately 100 mg of liver or other visceral tissue was homogenized in 500 μL of pre-chilled RIPA lysis buffer (Thermo Fisher) supplemented with protease inhibitor (Sigma) using three grinding beads (Beyotime) in a tissue homogenizer (65 Hz, 30 s, twice) at 4°C. The lysate was centrifuged at 13,000 rpm for 30 min at 4°C, and the supernatant was collected. Total protein concentration was determined using a BCA Protein Quantification Kit (YEASEN). RLN2 expression was measured using a Human Relaxin-2 Quantikine ELISA Kit (R&D Systems), type I collagen was quantified using a Mouse COL1 (Collagen Type I) ELISA Kit (Immunoway), and α-SMA levels were assessed using a Mouse ACTA2 (Actin alpha 2, Aortic Smooth Muscle) ELISA Kit (ABclonal). Protein concentrations were calculated by comparing sample absorbance values with standard curves.

### Protein analysis and quantification by western blot

Liver tissue samples were lysed in RIPA buffer (Thermo Fisher) containing protease inhibitor (Sigma). Protein concentration was determined using a BCA Protein Quantification Kit (YEASEN). Samples were diluted in 2× Laemmli buffer containing reducing agent and heated at 95°C for 5 min. Proteins were separated by 4%–20% SDS-PAGE (GenScript) and transferred to a 0.45 μm polyvinylidene fluoride membrane (Bio-Rad). The membrane was blocked with 5% BSA for 1 h at room temperature, followed by overnight incubation with primary antibodies at 4°C. After TBST washing, the membrane was incubated with secondary antibodies (appropriately diluted) for 2 h at room temperature. Protein bands were visualized using an ECL chemiluminescence substrate (Tanon), with GAPDH serving as a loading control. Images were captured using Image Lab 6.0.1, and band intensities were quantified using ImageJ 1.54 g. The antibodies used for the analysis are listed in Table S4.

### RNA quantification by qPCR

Total RNA was extracted from liver or 293T cells using TRIzol (Thermo Fisher). cDNA was synthesized from total RNA using ABScript Neo RT Master Mix for qPCR with gDNA Remover (ABclonal). Real-time PCR was performed using SYBR Green I (ABclonal) on a Roche LightCycler 480 system. All primer sequences are listed in Table S2. GAPDH was used as an endogenous control for normalization.

### RNA-seq

RNA sequencing (RNA-seq) reads were quality-trimmed and adapter-filtered using fastp (v0.23.2). Cleaned reads were aligned to the *Mus musculus* reference genome (GRCm39, gencode.vM36.annotation.gtf) using STAR (v.2.7.10a). Gene-level expression counts were generated using featureCounts (v.2.0.6) from the Subread package. Differential expression analysis was performed in R (v.4.3.2) using DESeq2 (v.1.42.0). Normalization and dispersion estimation were followed by Wald tests to identify significantly altered genes. Significance thresholds were set at an adjusted *p* value (Benjamini-Hochberg correction) < 0.01 and an absolute log2(fold change) > 2. Functional enrichment analysis of DEGs was conducted using the clusterProfiler (v.4.6.0) package, focusing on GO biological processes with mouse-specific annotations.

### Statistical analysis and reproducibility

In the figures involving comparisons for multiple group, one- or two-way ANOVA was performed and followed by Turkey test using GraphPad Prism 8.0.2. Exact *p* values are indicated in figures or Table S5. Statistical significance was defined as *p* < 0.05 (∗*p* < 0.05, ∗∗*p* < 0.01, ∗∗∗*p* < 0.001, ∗∗∗∗*p* < 0.0001, unless otherwise specified). To ensure reproducibility, all experiments were independently repeated with consistent results.

## Data and code availability

The data that support the key findings of this study are available within the article and supplemental information or upon request from the corresponding author, Z.W. Raw files associated with the RNA-seq study have been deposited in the NCBI BioProject: PRJNA1271696 (https://dataview.ncbi.nlm.nih.gov/object/PRJNA1271696?reviewer=angd6l0jvb307ptqt7552ft6ih). All analyzed data during this study have been deposited in the Figshare database (https://doi.org/10.6084/m9.figshare.29224991.v1).

## Acknowledgments

We thank Ms. Yun Jiang for helps in preparing the paper, and members of Wang lab for discussion and comments of this manuscript. This work is supported by 10.13039/501100001809National Natural Science Foundation of China (32030064 and 32250013 to Z.W.), the 10.13039/501100012166National Key Research and Development Program of China (2021YFA1300503 to Z.W), and the Strategic Priority Research Program of 10.13039/501100002367Chinese Academy of Sciences (XDB38040100 to Z.W.).

## Author contributions

Conceptualization, Z.W.; methodology, J.Z., Z.Z., Q.Z., L.X., C.W., Y.Y., and Z.W.; validation, Z.W.; formal analysis, J.Z., Q.Z., and L.X.; investigation, J.Z. and Q.Z.; resources, J.Z., Q.Z., and Y.Y.; data curation, J.Z., Z.Z., Q.Z., and L.X.; writing –original draft preparation, J.Z.; writing – review and editing, J.Z., Z.Z., Q.Z., and Z.W.; supervision, Z.W.; project administration, Z.W.; funding acquisition, Z.W.; Y.Y. and the CirCode BioMed team produced circRNAs and performed quality control of LNP-circRNA in the animal work. All authors have read and agreed to the published version of the manuscript.

## Declaration of interests

Z.W. and Y.Y. have co-founded a company, CirCode Biomedicine Inc., to commercialize the circRNAs therapeutics. Z.W. and Y.Y. submitted application for two patent families related to circRNA technology (CN111718929A and CN115404240A).

## Supplemental information

Document S1. Figures S1–S8 and Tables S1–S5

Document S2. Article plus supplemental information
