# Supplementary material for: Circular RNA encoding relaxin-2 as a potential therapy for liver fibrosis
Source: Mol Ther Nucleic Acids. 2025 Dec 18;37(1):102807. doi: 10.1016/j.omtn.2025.102807 (PMC12805276; doi:10.1016/j.omtn.2025.102807)
Supplement: Document S1. Figures S1–S8 and Tables S1–S5 [file mmc1.pdf]

OMTN, Volume *37*

Supplemental information

Circular RNA encoding relaxin-2 as a potential therapy for liver fibrosis

Jiewen Zhong, Zheyu Zhang, Lixing Xiao, Cheng Wang, Yun Yang, Qinghao Zhang, and Zefeng Wang

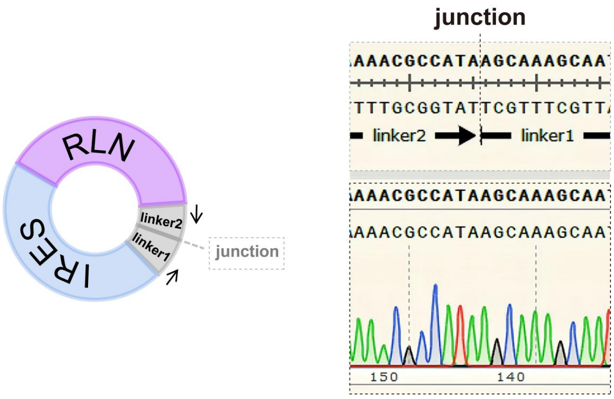

Figure S1 Validation of circRNA circularization. Sanger sequencing of the junction region after reverse transcription of purified cRLN2.

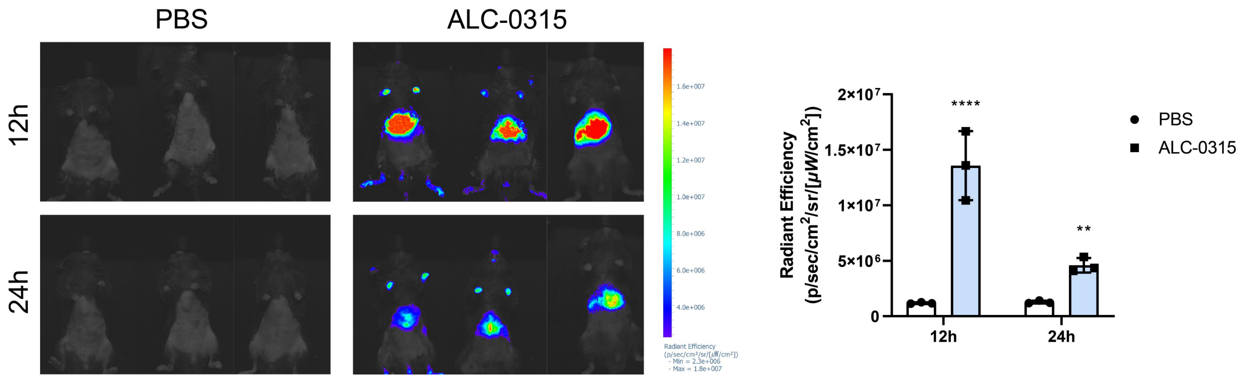

Figure S2 LNP selection of circRNAs. Liver delivery efficiency of LNP containing ALC-0315. The circRNAs encoding firefly luciferase (cFluc) was encapsulated with LNP and delivered into healthy mice through intravenous injection. The imaging was performed at 12h and 24h post-injection following intraperitoneal substrate administration, with fluorescence intensity quantified with IVIS Lumina system (PerkinElmer) (n=3).

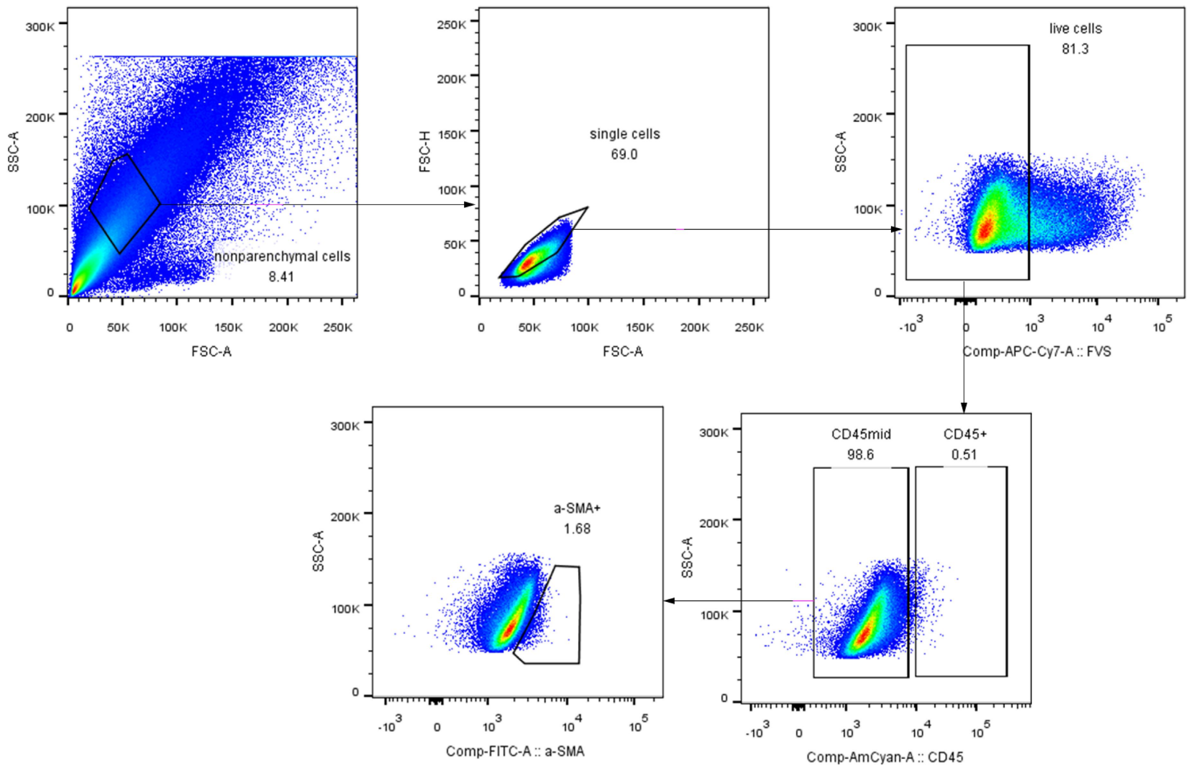

Figure S3 Gating strategy for α-SMA+ hepatic stellate cell isolation by FACS. Gating hierarchy for α-SMA+ HSCs (live CD45−Ly6G−CD11b−F4/80−Ly6C−Clec4f+α-SMA+ cells) from liver single-cell suspensions after Ficoll density gradient centrifugation.

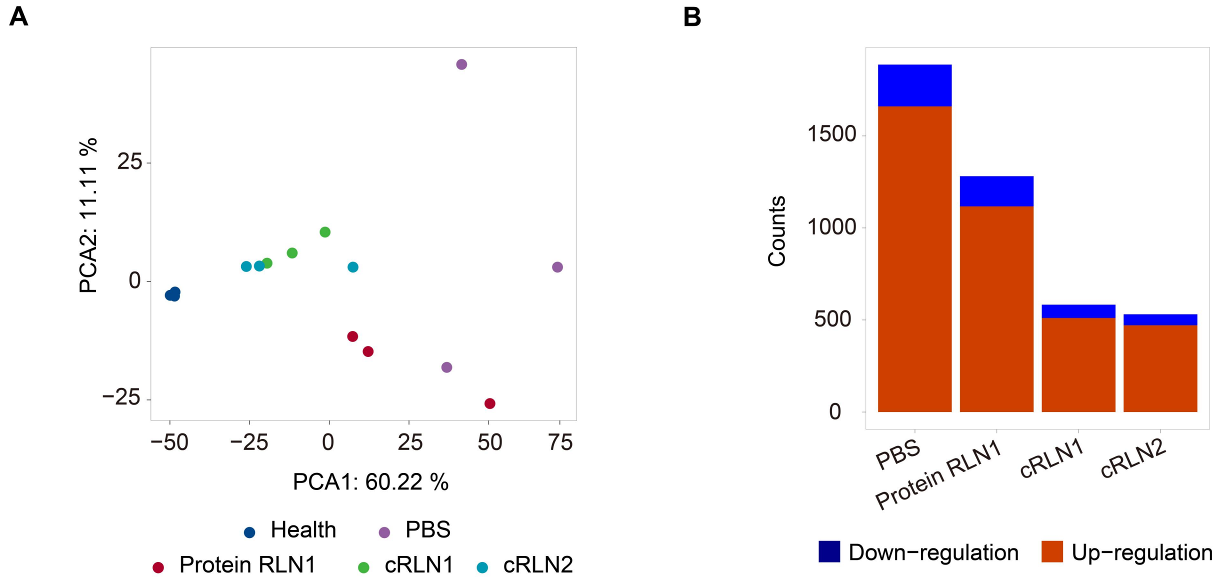

Figure S4 Differential gene expression analysis. (A) Principal component analysis (PCA) using all transcripts in the controls and treatment groups: Healthy, PBS, Protein RLN1, cRLN1, and cRLN2 (n=3 mice). (B) Bar plot showing numbers of DEGs between treatment groups and Healthy controls (n=3 mice).

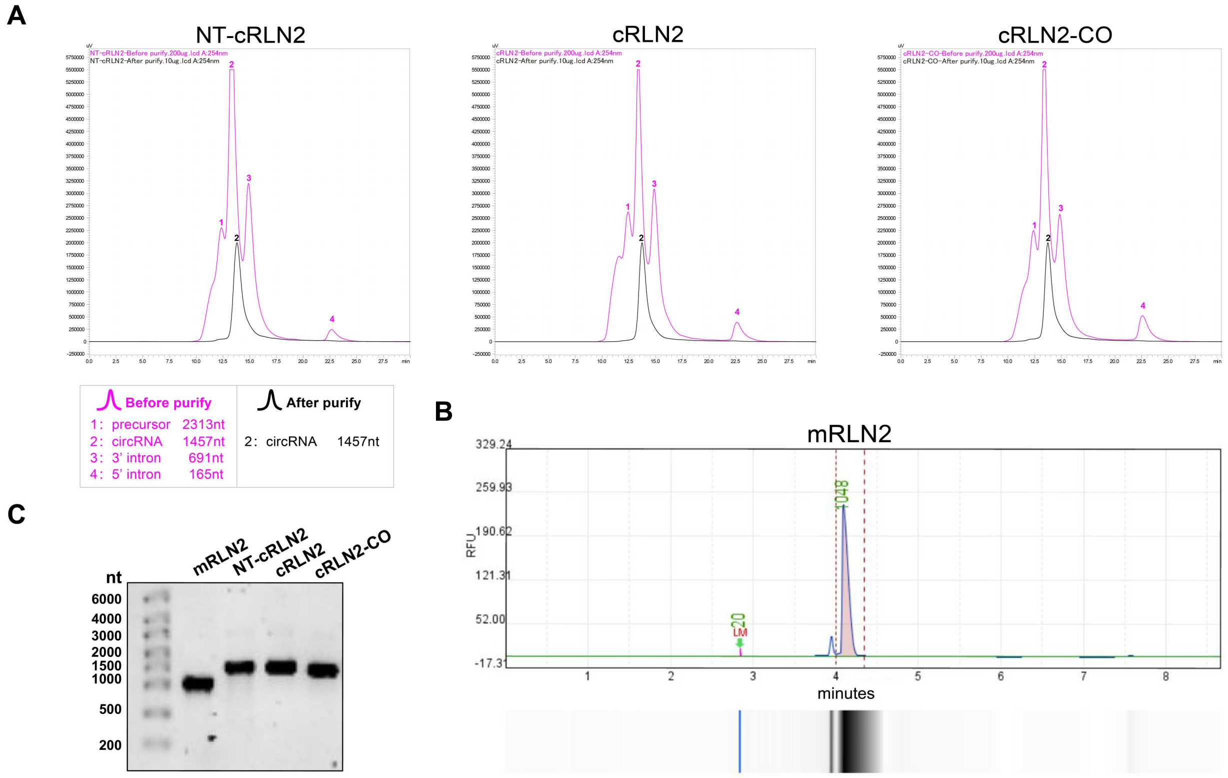

Figure S5 Preparation of human relaxin-expressing mRNA and circRNAs. (A) Representative HPLC chromatograms of circRNAs before and after purification. Pre-purification HPLC shows four major peaks: precursor (2313 nt), cRLN2 (1457 nt), 3' intron (691 nt), and 5' intron (165 nt). Post-purification HPLC displays a single peak matching the cRLN2 (1457 nt) stripe. (B) mRLN2 quality assessment using Qseq. (C) 1% low-melting agarose gel electrophoresis assessing RNAs purity.

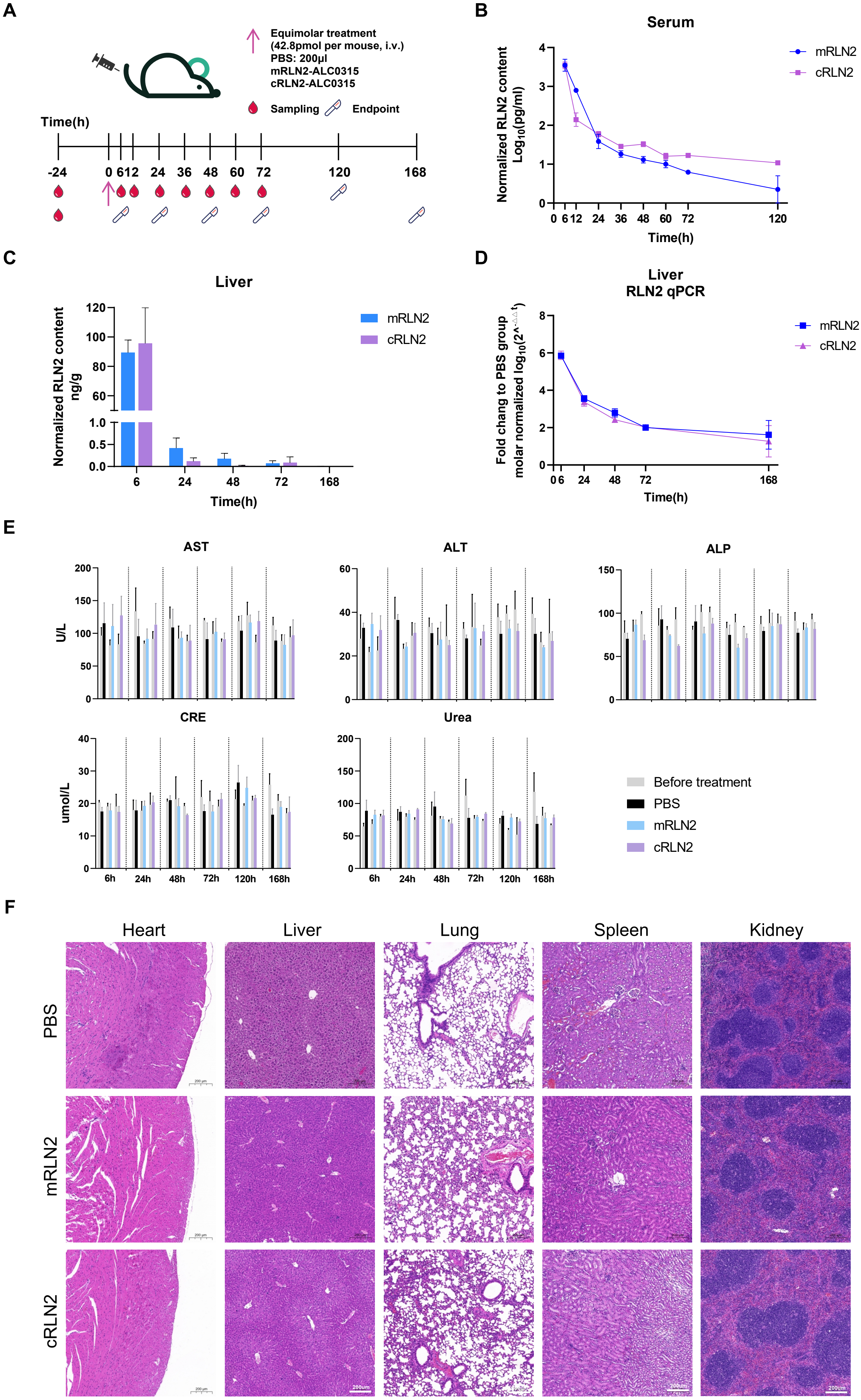

A
Equimolar treatment
(42.8pmol per mouse, i.v.)
PBS: 200μl
mRLN2-ALC0315
cRLN2-ALC0315
Sampling
Endpoint
Time(h)
-24
0 6 12 24 36 48 60 72 120 168
B
Serum
Normalized RLN2 content Log10(pg/ml)
mRLN2
cRLN2
Time(h)
C
Liver
Normalized RLN2 content ng/g
mRLN2
cRLN2
Time(h)
D
Liver
RLN2 qPCR
Fold chang to PBS group molar normalized log10(2^-ΔΔt)
mRLN2
cRLN2
Time(h)
E
AST
ALT
ALP
U/L
CRE
Urea
umol/L
6h 24h 48h 72h 120h 168h
Before treatment
PBS
mRLN2
cRLN2
F
Heart
Liver
Lung
Spleen
Kidney
PBS
mRLN2
cRLN2

Figure S6 Extended pharmacokinetic and biosafety comparison between mRLN2 and cRLN2. (A) Pharmacokinetic study design in healthy mice. After i.v. injection of mRLN2 or cRLN2 at 42.8 pmol dose, the blood and tissue samples were collected at different time points (n=3 mice). (B) RLN2 levels in serum over a span of 120h post-injection (n=3 mice). (C) RLN2 levels in liver over a span of 168h post-injection (n=3 mice). (D) Residual exogenous RNA in liver tissue by RT-qPCR using RLN2 CDS primers over a span of 168h post-injection (n=3 mice). (E) Serum ALT, AST, ALP, CRE, and Urea levels at baseline, 6h, 24h, 48h, 72h, 120h, and 168h post-injection, evaluating hepatorenal toxicity (n=3 mice). (F) H&E staining of major organs at 120h (heart, liver, spleen, lung, kidney).

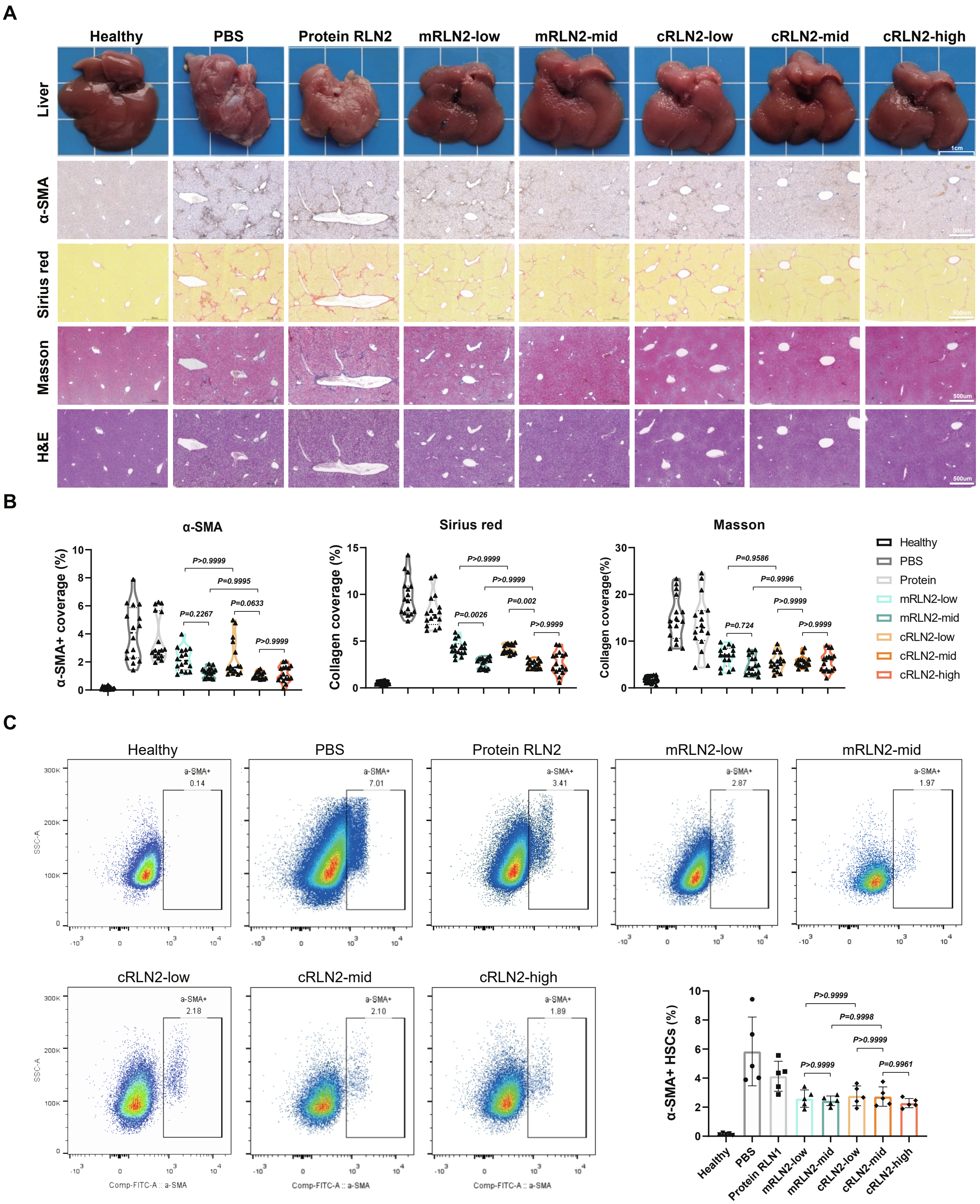
A
Healthy
PBS
Protein RLN2
mRLN2-low
mRLN2-mid
cRLN2-low
cRLN2-mid
cRLN2-high
Liver
α-SMA
Sirius red
Masson
H&E
1cm
500um
B
α-SMA
α-SMA+ coverage (%)
P>0.9999
P=0.9995
P=0.2267
P=0.0633
P>0.9999
Sirius red
Collagen coverage (%)
P>0.9999
P>0.9999
P=0.0026
P=0.002
P>0.9999
Masson
Collagen coverage(%)
P=0.9586
P=0.9996
P>0.9999
P=0.724
P>0.9999
Healthy
PBS
Protein
mRLN2-low
mRLN2-mid
cRLN2-low
cRLN2-mid
cRLN2-high
C
Healthy
PBS
Protein RLN2
mRLN2-low
mRLN2-mid
cRLN2-low
cRLN2-mid
cRLN2-high
a-SMA+
0.14
7.01
3.41
2.87
1.97
2.18
2.10
1.89
SSC-A
Comp-FITC-A :: a-SMA
α-SMA+ HSCs (%)
P>0.9999
P=0.9998
P>0.9999
P>0.9999
P=0.9961
Healthy
PBS
Protein RLN1
mRLN2-low
mRLN2-mid
cRLN2-low
cRLN2-mid
cRLN2-high

Figure S7 Supplementary results of dose-response studies of mRLN2 and cRLN2. (A) Liver morphology and histology (α-SMA IHC, Sirius Red, Masson's trichrome, H&E) from Healthy, PBS, Protein RLN2, mRLN2-low, mRLN2-mid, cRLN2-low, cRLN2-mid, and cRLN2-high groups. Scale bars: 1 cm for liver images, 500 μm for histology. (B) Quantification of histology from four random fields per mouse (n=4 mice). (C) Flow cytometry analysis of α-SMA+ HSCs, quantifying activation status (n=5 mice). Experiments were repeated twice independently with similar results. Significant differences were assessed using a one-way ANOVA with Turkey test (B-C). Results are presented as mean ± s.d. from the second repeat. The p-values for the comparison between each treatment group and the PBS group are shown in Table S5.

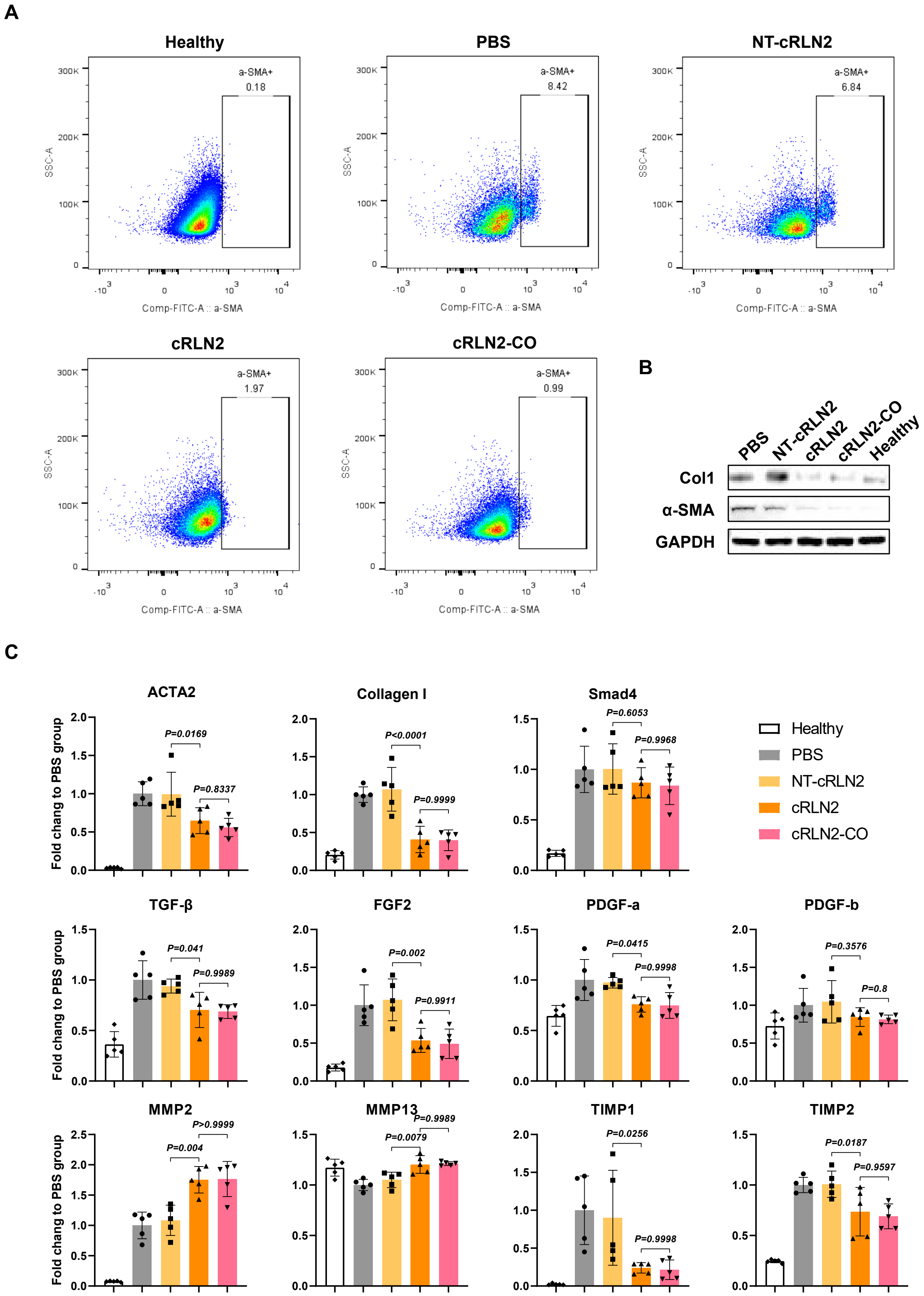
A
Healthy
PBS
NT-cRLN2
cRLN2
cRLN2-CO
a-SMA+
0.18
8.42
6.84
1.97
0.99
SSC-A
Comp-FITC-A :: a-SMA
B
PBS
NT-cRLN2
cRLN2
cRLN2-CO
Healthy
Col1
α-SMA
GAPDH
C
ACTA2
Collagen I
Smad4
TGF-β
FGF2
PDGF-a
PDGF-b
MMP2
MMP13
TIMP1
TIMP2
Fold chang to PBS group
P=0.0169
P=0.8337
P<0.0001
P=0.9999
P=0.6053
P=0.9968
P=0.041
P=0.9989
P=0.002
P=0.9911
P=0.0415
P=0.9998
P=0.3576
P=0.8
P=0.004
P>0.9999
P=0.0079
P=0.9989
P=0.0256
P=0.9998
P=0.0187
P=0.9597
Healthy
PBS
NT-cRLN2
cRLN2
cRLN2-CO

Figure S8 Supplementary results of cRLN2 sequence optimization for liver fibrosis treatment. (A) Representative FACS analysis of α-SMA+ HSCs across treatment groups. (B) Western blot of hepatic α-SMA and collagen I expression. (C) Relative mRNA levels of fibrotic markers (*ACTA2*, *Collagen I*, *TGF-β*, *FGF2*, *PDGF-a/b*, *MMP2/9*, *TIMP1/2*, and *Smad4*) in Healthy, PBS, NT-cRLN2, cRLN2, and cRLN2-CO groups (n=5 mice). Experiments were repeated twice independently with similar results. Significant differences were assessed using a one-way ANOVA with Turkey test (C). Results are presented as mean ± s.d. from the second repeat. The p-values for the comparison between each treatment group and the PBS group are shown in Table S5.

Table S1. Characterizations of RNA-LNPs

Name	Size(nm)	PDI	Zeta Potential (mV)	EE(%)
cFluc-ALC0315	123.6±1.36	0.01±0.011	-2.9	88%
cRLN1-ALC0315	165.1±0.68	0.06±0.016	-9.5	82%
cRLN2-ALC0315	82.0±1.61	0.24±0.042	-14.5	86%
mRLN2-ALC0315	81.0±1.81	0.22±0.019	-7.7	83%
NT-RLN2-ALC0315	74.9±0.20	0.18±0.004	-7.4	87%
cRLN2-CO-ALC0315	100.1±9.29	0.40±0.040	-11.2	91%

Table S2. qPCR primers

Target	Forward	Reverse
Mouse *GAPDH*	AGAAGGTGGTGAAGCAGGCATC	CGAAGGTGGAAGAGTGGGAGTTG
Mouse *TGF-β*	GAGAAGAACTGCTGTGTGCG	GTGTCCAGGCTCCAAATATAG
Mouse *FGF2*	CTACAACTCCAAGCAGAAGAG	TTAGAAGCCAGCAGCCGTC
Mouse *PDGF-a*	GACCAGGACGGTCATTTACG	TGGTGTTACAACAGCCAGTG
Mouse *PDGF-b*	CCACCCTCTAGCTTCGTTGC	GGCTCGGGTCAGTCTGTCTA
Mouse *MMP2*	GTCTCAAGAGCGTGAAGTTTGGAAG	ACCTGGTGTGCAGCGATGAAG
Mouse *MMP9*	CCTGTGTGTTCCCGTTCATCTTTG	TTATCCTGGTCATAGTTGGCTGTGG
Mouse *MMP13*	TCATACTACCATCCTGCGACTCTTG	TGCCAGTCACCTCTAAGCCAAAG
Mouse *TIMP1*	TTCAAGGCTGTGGGAAATGC	CCACAGCCAGCACTATAGGT
Mouse *TIMP2*	CAACGAGACCCTGCAGTCTA	GAAATCAGCAGAAACAGGCA
Mouse *ACTA2*	GTACCACCATGTACCCAGGC	GCTGGAAGGTAGACAGCGAA
Mouse *Collagen I*	AGCACGTCTGGTTTGGAGAG	GACATTAGGCGCAGGAAGGT
Mouse *Smad4*	AAGTCAGCCGGCCAGTATTC	CAGTCCAGGTGGTAGTGCTG
RLN2	AATTAGTTCGCGCGCAGATT	AGCTCCTGTGGCAAATTAGC
circRNA junction	GATCTCTTGCTAGATTTTGC	CAACCCACAGGCTGTTTTAA
Human *GAPDH*	TGAGTACGTCGTGGAGTCCA	CACACCCATGACGAACATGG

Table S3. Sequence

Name	Sequence
mRLN2 5'UTR	CAAAAGACCGCTTGAGCCGGGTAGGGAAAGCAGCCTAAAGCCCGGGACAG<br>GCACACAGGCCCAGGTGTGTAGGCCACAGCAGCTGCAGTCCTGAAAGGCT<br>GCAACGTTCCGACCTCCAGGAGAGACCAGGCCCAGG
mRLN2 CDS	ATGCCTCGCCTGTTTTTTTTCCACCTGCTAGGAGTCTGTTTACTACTGAACCA<br>ATTTTCCAGAGCAGTCGCGGACTCATGGATGGAGGAAGTTATTAAATTATGCG<br>GCCGCGAATTAGTTCGCGCGCAGATTGCCATTTGCGGCATGAGCACCTGGA<br>GCAAAAGGTCTCTGAGCCAGGAAGATGCTCCTCAGACACCTAGACCAGTGG<br>CAGAAATTGTGCCATCCTTCATCAACAAAGATACAGAAACCATAAATATGATGT<br>CAGAATTTGTTGCTAATTTGCCACAGGAGCTGAAGTTAACCCTGTCTGAGATG<br>CAGCCAGCATTACCACAGCTACAACAACATGTACCTGTATTAAAAGATTCCAG<br>TCTTCTCTTTGAAGAATTTAAGAAACTTATTCGCAATAGACAAAGTGAAGCCG<br>CAGACAGCAGTCCTTCAGAATTAAAATACTTAGGCTTGGATACTCATTCTCGA<br>AAAAAGAGACAACTCTACAGTGCATTGGCTAATAAATGTTGCCATGTTGGTTG<br>TACCAAAAGATCTCTTGCTAGATTTTGCTGA
mRLN2 3'UTR	GATGAAGCTAATTGTGCACATCTCGTATAATATTCACACATATTCTTAATGACAT<br>TTCACTGATGCTTCTATCAGGTCCCATCAATTCTTAGAATATCTAAGAATCTTTG<br>TTAGATATTAGGTCCCATCAATTCTTAGAATATCTAAACATCTTTGTTGATGTTTA<br>GATTTTTTTATTTGATGTGTAAGAAAATGTTCTTTGTGTGATTAAATGACACATT<br>TTTTTGCTGAAAAGCTAGCAAAAAAAAAAAAAAAAAAAAAAAAAAAAAAGCATA<br>TGACTAAAAAAAAAAAAAAAAAAAAAAAAAAAAAAAAAAAAAAAAAAAAA<br>AAAAAAAAAAAAAAAAAAAAA
CVB3 IRES	TTAAAACAGCCTGTGGGTTGATCCCACCCACAGGGCCCATTGGGCGCTAGC<br>ACTCTGGTATCACGGTACCTTTGTGCGCCTGTTTTATACCCCCTCCCCCAACT<br>GTAACTTAGAAGTAACACACACCGATCAACAGTCAGCGTGGCACACCAGCCA<br>CGTTTTGATCAAGCACTTCTGTTACCCCGGACTGAGTATCAATAGACTGCTCA<br>CGCGGTTGAAGGAGAAAGCGTTCGTTATCCGGCCAACTACTTCGAAAAACCT<br>AGTAACAACGTGGAAGTTGCAGAGTGTTTCGCTCAGCACTACCCCAGTGTAG<br>ATCAGGTCGATGAGTCACCGCATTCCCCACGGGCGACCGTGGCGGTGGCTG<br>CGTTGGCGGCCTGCCCATGGGGAAACCCATGGGACGCTCTAATACAGACAT<br>GGTGCGAAGAGTCTATTGAGCTAGTTGGTAGTCCTCCGGCCCCTGAATGCG<br>GCTAATCCTAACTGCGGAGCACACACCCTCAAGCCAGAGGGCAGTGTGTCG<br>TAACGGGCAACTCTGCAGCGGAACCGACTACTTTGGGTGTCCGTGTTTCATT<br>TTATTCCTATACTGGCTGCTTATGGTGACAATTGAGAGATTGTTACCATATAGCT<br>ATTGGATTGGCCATCCGGTGACCAATAGAGCTATTATATATCTCTTTGTTGGGT<br>TTATACCACTTAGCTTGAAAGAGGTTAAAACATTACAATTCATTGTTAAGTTGAA<br>TACAGCAAA

cRLN2 CDS

ATGCCTCGCCTGTTTTTTTTCCACCTGCTAGGAGTCTGTTTACTACTGAACCA
ATTTTCCAGAGCAGTCGCGGACTCATGGATGGAGGAAGTTATTAAATTATGCG
GCCGCGAATTAGTTCGCGCGCAGATTGCCATTTGCGGCATGAGCACCTGGA
GCAAAAGGTCTCTGAGCCAGGAAGATGCTCCTCAGACACCTAGACCAGTGG
CAGAAATTGTGCCATCCTTCATCAACAAAGATACAGAAACCATAAATATGATGT
CAGAATTTGTTGCTAATTTGCCACAGGAGCTGAAGTTAACCCTGTCTGAGATG
CAGCCAGCATTACCACAGCTACAACAACATGTACCTGTATTAAAAGATTCCAG
TCTTCTCTTTGAAGAATTTAAGAAACTTATTCGCAATAGACAAAGTGAAGCCG
CAGACAGCAGTCCTTCAGAATTAAAATACTTAGGCTTGGATACTCATTCTCGA
AAAAAGAGACAACTCTACAGTGCATTGGCTAATAAATGTTGCCATGTTGGTTG
TACCAAAAGATCTCTTGCTAGATTTTGCTGA

NT-cRLN2 CDS

TAACCTCGCCTGTTTTTTTTCCACCTGCTAGGAGTCTGTTTACTACTGAACCA
ATTTTCCAGAGCAGTCGCGGACTCATGGATGGAGGAAGTTATTAAATTATGCG
GCCGCGAATTAGTTCGCGCGCAGATTGCCATTTGCGGCATGAGCACCTGGA
GCAAAAGGTCTCTGAGCCAGGAAGATGCTCCTCAGACACCTAGACCAGTGG
CAGAAATTGTGCCATCCTTCATCAACAAAGATACAGAAACCATAAATATGATGT
CAGAATTTGTTGCTAATTTGCCACAGGAGCTGAAGTTAACCCTGTCTGAGATG
CAGCCAGCATTACCACAGCTACAACAACATGTACCTGTATTAAAAGATTCCAG
TCTTCTCTTTGAAGAATTTAAGAAACTTATTCGCAATAGACAAAGTGAAGCCG
CAGACAGCAGTCCTTCAGAATTAAAATACTTAGGCTTGGATACTCATTCTCGA
AAAAAGAGACAACTCTACAGTGCATTGGCTAATAAATGTTGCCATGTTGGTTG
TACCAAAAGATCTCTTGCTAGATTTTGCTGA

cRLN2-CO CDS

ATGCCCAGACTGTTTTTCTTCCATCTGCTCGGCGTGTGCCTGCTGCTGAACC
AGTTCAGCAGAGCCGTGGCCGATTCTTGGATGGAAGAAGTGATCAAGCTGT
GTGGCAGAGAGCTGGTGCGGGCCCAGATCGCCATCTGCGGCATGTCCACCT
GGTCCAAGCGGAGCCTTTCTCAGGAGGACGCCCCTCAGACCCCTAGACCTG
TCGCTGAAATCGTGCCCAGCTTCATCAACAAAGATACAGAGACAATCAACATG
ATGAGCGAGTTCGTGGCCAATCTGCCCCAAGAGCTGAAACTGACCCTGAGC
GAAATGCAGCCTGCTCTGCCTCAACTGCAGCAGCACGTGCCAGTGCTGAAG
GACTCTAGCCTGCTGTTCGAGGAATTTAAGAAGCTGATTAGAAACAGACAGA
GCGAGGCCGCCGACAGCAGCCCTAGCGAGCTGAAGTACCTGGGCCTGGAC
ACCCACAGCCGGAAGAAACGGCAGCTGTACAGCGCCCTGGCCAACAAGTG
CTGCCACGTTGGATGTACCAAGCGCTCCCTGGCTAGATTCTGCTAA

Table S4. Antibody information

Name	Supplier	Catalog #	Application	Dilution
CD45-BV510	BioLegend	103137	Flow	1:100
Ly6G-Pacific blue	BioLegend	127611	Flow	1:100
CD11b-AF700	BioLegend	101222	Flow	1:100
F4/80-PE	BioLegend	123109	Flow	1:100
Ly6C-PE Cy7	BioLegend	128017	Flow	1:100
Clecf-AF647	BioLegend	156803	Flow	1:100
aSMA-AF488	Invitrogen	53-9760-82	Flow	1:100
Anti-α-SMA	Boster	M01072-1	IHC	1:1000
Anti-α-SMA	Abcam	Ab7817	WB	1:500
Anti-Collagen I	Abcam	Ab34710	WB	1:500
tgfb1	Abclone	A23262	WB	1:500
Phospho-SMAD2	CST	#3108	WB	1:1000
SMAD2/3	CST	#3102	WB	1:1000
HRP-conjugated GAPDH Mouse mAb	Abclone	AC035	WB	1:4000
HRP-conjugated goat anti-mouse IgG	Abcam	Ab6789	IHC	1:1000
Goat Anti-rabbit IgG	CST	7074S	WB	1:1000
Horse Anti-mouse IgG	CST	7076S	WB	1:1000

Table S5. PBS vs treatment group *P* value

Figure 2	2F		2G	2H				2I
	AST	ALT	Ultrasound	TGF-β	pSmad2	col I	α-SMA	cAMP
**Protein RLN1**	0.6455	0.24	<0.0001	0.0191	0.0302	0.9974	0.9953	0.9842
**cRLN1**	<0.0001	<0.0001	<0.0001	0.0034	0.0003	0.0333	0.025	0.0538
**cRLN2**	<0.0001	<0.0001	<0.0001	0.0019	0.0003	0.0178	0.0226	0.0021

2J			2K			2L		
IL-6	TNF-α	IFN-α	HYP	col I	α-SMA	α-SMA	SR	Masson
0.0188	0.8439	0.0085	0.076	0.9848	0.4034	0.2862	0.0729	0.0322
<0.0001	<0.0001	<0.0001	0.0006	0.0172	0.0971	<0.0001	<0.0001	<0.0001
<0.0001	<0.0001	<0.0001	0.0015	0.0569	0.6631	<0.0001	<0.0001	<0.0001

Figure 3	3A		3B					
	aHSCs	ACTA2	col I	TGF-β	FGF	PDGF-a	PDGF-b	MMP2
**Protein RLN1**	0.1046	0.4022	<0.0001	0.0007	0.8301	0.0553	0.131	0.3171
**cRLN1**	<0.0001	<0.0001	<0.0001	<0.0001	<0.0001	<0.0001	<0.0001	0.8364
**cRLN2**	0.0002	<0.0001	<0.0001	<0.0001	<0.0001	<0.0001	0.0005	0.8857

3B		
MMP9	TIMP1	TIMP2
0.9999	0.8593	0.7597
0.2494	0.0238	<0.0001
>0.9999	0.0003	0.0006

Figure 5	5B		5D	5E		5F		
	AST	ALT	Ultrasound	col I	α-SMA	HYP	col I	α-SMA
**Protein RLN2**	<0.0001	0.0195	<0.0001	0.581	<0.0001	0.0108	0.0002	0.001
**mRLN2-low**	<0.0001	<0.0001	<0.0001	0.0023	<0.0001	<0.0001	<0.0001	<0.0001
**mRLN2-mid**	<0.0001	<0.0001	<0.0001	0.0001	<0.0001	<0.0001	<0.0001	<0.0001
**cRLN2-low**	<0.0001	<0.0001	<0.0001	0.0033	<0.0001	0.0068	<0.0001	<0.0001
**cRLN2-mid**	<0.0001	<0.0001	<0.0001	0.0001	<0.0001	<0.0001	<0.0001	<0.0001
**cRLN2-high**	<0.0001	<0.0001	<0.0001	0.0002	<0.0001	<0.0001	<0.0001	<0.0001

5G								
ACTA2	col I	TGF-β	FGF	PDGF-a	PDGF-b	MMP2	MMP13	TIMP1
0.001	<0.0001	0.0296	<0.0001	0.6146	0.8041	0.0311	0.3593	0.2798
<0.0001	<0.0001	0.0014	<0.0001	0.1428	0.1318	0.0012	0.0709	0.0017
<0.0001	<0.0001	0.0005	<0.0001	0.0113	0.0015	<0.0001	0.0281	0.0007
0.0001	<0.0001	0.0101	<0.0001	0.0951	0.2371	0.0326	0.3084	0.0039
<0.0001	<0.0001	0.0015	<0.0001	0.0022	0.0046	0.0007	0.0016	0.0011
<0.0001	<0.0001	0.0012	<0.0001	0.0619	0.0072	0.005	0.0352	0.002

Figure 5	5G	
	TIMP2	Smad4
**Protein RLN2**	0.4876	<0.0001
**mRLN2-low**	0.0409	<0.0001
**mRLN2-mid**	0.0013	<0.0001
**cRLN2-low**	0.1435	<0.0001
**cRLN2-mid**	0.002	<0.0001
**cRLN2-high**	0.0704	<0.0001

Figure 6	6B		6C		6D		6E	6F		
	AST	ALT	Ultrasound	HYP	col I	α-SMA	aHSCs	α-SMA	SR	Masson
**NT-cRLN2**	0.8415	0.9997	0.9965	0.8574	0.962	0.9598	0.9453	0.7375	0.7381	0.0665
**cRLN2**	<0.0001	<0.0001	<0.0001	0.0053	0.002	0.1345	<0.0001	<0.0001	<0.0001	<0.0001
**cRLN2-CO**	<0.0001	<0.0001	<0.0001	0.0116	0.0017	0.0852	<0.0001	<0.0001	<0.0001	<0.0001

Figure S7		S7B		S7C
	α-SMA	SR	Masson	aHSCs
**Protein RLN2**	0.8024	0.0013	0.8414	0.0612
**mRLN2-low**	<0.0001	<0.0001	<0.0001	0.0001
**mRLN2-mid**	<0.0001	<0.0001	<0.0001	<0.0001
**cRLN2-low**	<0.0001	<0.0001	<0.0001	0.0003
**cRLN2-mid**	<0.0001	<0.0001	<0.0001	0.0002
**cRLN2-high**	<0.0001	<0.0001	<0.0001	<0.0001

Figure S8	S8C								S8C		
	ACTA2	col I	TGF-β	FGF	PDGF-a	PDGF-b	MMP2	MMP13	TIMP1	TIMP2	Smad4
**NT-cRLN2**	>0.9999	0.91	0.89	0.949	0.99	0.7033	0.932	0.6069	0.9755	0.9999	>0.9999
**cRLN2**	0.0149	<0.0001	0.0089	0.0071	0.0197	0.3664	0.0001	0.0006	0.0096	0.0226	0.6285
**cRLN2-CO**	0.0024	<0.0001	0.0058	0.0033	0.0148	0.3464	<0.0001	0.0004	0.0075	0.0073	0.4608
